# Activity-Dependent Inhibitory Synapse Scaling Is Determined by Gephyrin Phosphorylation and Subsequent Regulation of GABA_A_ Receptor Diffusion

**DOI:** 10.1523/ENEURO.0203-17.2017

**Published:** 2018-01-18

**Authors:** Sereina Battaglia, Marianne Renner, Marion Russeau, Etienne Côme, Shiva K. Tyagarajan, Sabine Lévi

**Affiliations:** 1 INSERM UMR-S, Paris, 75005, France; 2 Université Pierre et Marie Curie, Paris, 75005, France; 3 Institut du Fer a Moulin, Paris, 75005, France; 4 Institute of Pharmacology and Toxicology, University of Zürich, Zurich, 8057, Switzerland; 5 Center for Neuroscience Zurich, Zurich, 8057, Switzerland

**Keywords:** GABAA receptor, homeostatic plasticity, PALM, post-translation modification, single particle tracking

## Abstract

Synaptic plasticity relies on the rapid changes in neurotransmitter receptor number at postsynaptic sites. Using superresolution photoactivatable localization microscopy imaging and quantum dot–based single-particle tracking in rat hippocampal cultured neurons, we investigated whether the phosphorylation status of the main scaffolding protein gephyrin influenced the organization of the gephyrin scaffold and GABA_A_ receptor (GABA_A_R) membrane dynamics. We found that gephyrin phosphorylation regulates gephyrin microdomain compaction. Extracellular signal–regulated kinase 1/2 and glycogen synthase kinase 3β (GSK3β) signaling alter the gephyrin scaffold mesh differentially. Differences in scaffold organization similarly affected the diffusion of synaptic GABA_A_Rs, suggesting reduced gephyrin receptor–binding properties. In the context of synaptic scaling, our results identify a novel role of the GSK3β signaling pathway in the activity-dependent regulation of extrasynaptic receptor surface trafficking and GSK3β, protein kinase A, and calcium/calmodulin-dependent protein kinase IIα pathways in facilitating adaptations of synaptic receptors.

## Significance Statement

Our data identify phosphorylation as a key mechanism controlling the gephyrin scaffold mesh, and hence, the diffusion capture of GABA_A_ receptors at inhibitory synapses. We further show how critical this mechanism is for inhibitory synaptic scaling.

## Introduction

Fast synaptic inhibition mediated by GABA_A_ receptors (GABA_A_Rs) plays an essential role in information transfer between neurons. In recent years GABAergic inhibition has been shown to be dynamic, allowing flexible adaptations (Chen et al., 2012). Within the paradigm of *in vitro* synaptic scaling, wherein the neuronal activity is pharmacologically manipulated for several hours to days, the effects of chronic changes in activity are still poorly understood at inhibitory synapses.

Neuronal inhibition is dynamically regulated by the amount of network activity. GABA_A_R stability at synaptic sites and subsequent proteasomal degradation is an essential component of synaptic homeostasis that strongly influences amplitude and frequency of miniature inhibitory postsynaptic currents (mIPSCs; Saliba et al., 2007). Similarly, lasting depolarization decreases GABA_A_R internalization on principal neurons and increases GAD65 cluster size at presynaptic GABAergic terminals (Rannals and Kapur, 2011). These observations highlight that multiple systems and pathways facilitate inhibitory synapse adjustments in response to chronic changes in activity.

At postsynaptic sites, lateral diffusion in and out of synapses can also rapidly alter receptor availability on acute activity elevation (Bannai et al., 2009, 2015). Chemical-induced long-term potentiation (iLTP) enhances phosphorylation of the GABA_A_R β3 subunit at serine 383 (S383) by calcium/calmodulin-dependent protein kinase IIα (CaMKIIα), resulting in reduced surface mobility of GABA_A_Rs, synaptic enrichment of receptors, and increased inhibitory neurotransmission (Petrini et al., 2014). Hence, apart from endocytosis and exocytosis, lateral diffusion of receptors could also be an effective mechanism of synaptic plasticity.

In recent years, it has become evident that the main scaffolding protein at the GABAergic synapse, gephyrin, is dynamically regulated, and this contributes to input-specific adaptations at postsynaptic sites (Chen et al., 2012; van Versendaal et al., 2012; Villa et al., 2016). Identification of signaling pathways that converge onto gephyrin scaffolds by causing posttranslational modifications of specific residues has shed new light on the molecular mechanisms underlying GABAergic synaptic plasticity. It was revealed that gephyrin phosphorylation by extracellular signal–regulated kinase 1/2 (ERK1/2) at serine 268 (S268) reduces scaffold size and GABAergic mIPSC amplitude (Tyagarajan et al., 2013). Similarly, blocking glycogen synthase kinase 3β (GSK3β) phosphorylation of gephyrin at serine 270 via the transgenic expression of the phospho-null mutant (S270A) significantly increases mIPSC frequency and amplitude (Tyagarajan et al., 2011). Theta burst stimulation (TBS) of CA3 Schaffer collaterals has been reported to induce gephyrin-mediated remodeling of GABAergic synapses in CA1 pyramidal cells (Flores et al., 2015). Although gephyrin phosphorylation at CaMKIIα sites is involved in this form of structural plasticity (Flores et al., 2015), the molecular basis for gephyrin phosphorylation–induced GABA_A_R synapse dynamics remains to be further explored.

To address this, we rendered gephyrin insensitive to ERK1/2 and GSK3β signaling pathways and studied their influence on GABA_A_R membrane diffusion properties. We report structural organization differences within gephyrin scaffolds based on phosphorylation status. Furthermore, cooperation between gephyrin and GABA_A_Rs is differentially regulated by gephyrin phosphorylation status and changes in activity.

## Material and Methods

### Neuronal culture

Primary cultures of hippocampal neurons were prepared from hippocampi dissected at embryonic day 18 or 19 from Sprague-Dawley rats of either sex. Tissue was trypsinized (0.25% vol/vol) and mechanically dissociated in 1× HBSS (Invitrogen) containing 10 mM Hepes (Invitrogen). Neurons were plated at a density of 120 × 10^3^ cells/ml onto 18-mm-diameter glass coverslips (Assistent) precoated with 50 µg/ml poly-d,l-ornithine (Sigma-Aldrich) in plating medium composed of minimum essential medium (MEM, Sigma-Aldrich) supplemented with horse serum (10% vol/vol, Invitrogen), l-glutamine (2 mM), and Na^+^ pyruvate (1 mM; Invitrogen). After attachment for 3–4 h, cells were incubated in culture medium that consists of Neurobasal medium supplemented with B27 (1×), l-glutamine (2 mM), and antibiotics (penicillin 200 units/ml, streptomycin, 200 µg/ml; Invitrogen) for up to 4 wk at 37°C in a 5% CO_2_ humidified incubator. Each week, one-fifth of the culture medium volume was replaced.

### DNA constructs

The following constructs were used: *GEPHN* 3′-UTR shRNA and control shRNA-3m (Yu et al., 2007), DsRed-homer1c (Bats et al., 2007; provided by D. Choquet, IIN, Bordeaux, France), eGFP-gephyrin P1 variant (Lardi-Studler et al., 2007). eGFP- or pDendra2-WT, -S268E, -S270A, -DN, -S303A/S305A (SSA), and –SSA/S270A point mutants were generated using the eGFP-gephryin P1 variant as template for site-directed mutagenesis (Tyagarajan et al., 2011, 2013; Flores et al., 2015).

### Neuronal transfection

Transfections were conducted at 14–15 days *in vitro* (DIV) using Lipofectamine 2000 (Invitrogen) or Transfectin (Bio-Rad), according to the manufacturers’ instructions (DNA:transfectin ratio 1 µg:3 µl), with 1–1.2 µg of plasmid DNA per 20-mm well. The following ratio of plasmid DNA was used in cotransfection experiments: 0.5:0.5:0.3 μg for eGFP-S268E/eGFP-S270A/eGFP-DN/eGFP-SSA/eGFP-SSA/S270A:*GEPHN* 3′ UTR shRNA/*GEPHN* 3′ UTR-3m shRNA:DsRed-homer1c. Experiments were performed 6–9 d after transfection.

### Pharmacology

4-Aminopyridine (4-AP, 100 mM, Sigma-Aldrich) was directly added to the culture medium, and the neurons were returned to a 5% CO_2_ humidified incubator for 8 or 48 h before use. For SPT experiments, neurons were labeled at 37°C in imaging medium (see below for composition) in the presence of 4-AP, transferred to a recording chamber, and recorded within 45 min at 31°C in imaging medium in the presence of 4-AP. The imaging medium consisted of phenol red–free MEM supplemented with glucose (33 mM; Sigma-Aldrich) and Hepes (20 mM), glutamine (2 mM), Na^+^-pyruvate (1 mM), and B27 (1×) from Invitrogen.

### Immunocytochemistry

Cells were fixed for 15 min at room temperature (RT) in paraformaldehyde (PFA, 4% wt/vol, Sigma-Aldrich) and sucrose (14% wt/vol, Sigma) solution prepared in PBS (1×). After washes in PBS, cells were permeabilized with Triton (0.25% vol/vol, Sigma-Aldrich) diluted in PBS. Cells were washed again in PBS and incubated for 1 h at RT in Triton (0.1% vol/vol, Sigma-Aldrich) and goat serum (GS, 10% vol/vol, Invitrogen) in PBS to block nonspecific staining. Subsequently, neurons were incubated for 1 h with a primary antibody mix consisting of guinea pig antibodies against GABA_A_R α2 subunit (1:2000, provided by J.M. Fritschy, University of Zurich) and rabbit anti-VGAT (1:400, provided by B. Gasnier, University Paris Descartes, Paris) in PBS supplemented with GS (10% vol/vol, Invitrogen) and Triton (0.1% vol/vol, Sigma-Aldrich). After washes, cells were incubated for 60 min at RT with a secondary antibody mix containing biotinylated F(ab′)2 anti-guinea pig (1:300, Jackson Immunoresearch) and AMCA350-conjugated goat anti-rabbit (1:100, Jackson Laboratory) in PBS-GS-Triton blocking solution, washed, incubated for another 45 min with streptavidin-CY5 (1:300, Thermo Fisher Scientific), and finally mounted on glass slides using Mowiol 4-88 (48 mg/ml, Sigma-Aldrich). Sets of neurons compared for quantification were labeled simultaneously.

### Fluorescence image acquisition and analysis

Image acquisition was performed using a 63× objective (NA 1.32) on a Leica DM6000 upright epifluorescence microscope with a 12-bit cooled CCD camera (Micromax, Roper Scientific) run by MetaMorph software (Roper Scientific). Quantification was performed using MetaMorph software. Image exposure time was determined on bright cells to obtain the best fluorescence-to-noise ratio and avoid pixel saturation. All images from a given culture were then acquired with the same exposure time and acquisition parameters. For each image, several dendritic regions of interest were manually chosen, and a user-defined intensity threshold was applied to select clusters and avoid their coalescence. For quantification of gephyrin or GABA_A_R α2 synaptic clusters, gephyrin or receptor clusters comprising at least 3 pixels and colocalized on at least 1 pixel with VGAT clusters were considered. The integrated fluorescence intensities of clusters were measured.

### Live-cell staining for single-particle imaging

Neurons were incubated for 3–5 min at 37°C with primary antibodies against extracellular epitopes of GABA_A_R α2 subunit (guinea pig, 1:750/1:1000, provided by J.M. Fritschy), washed, and incubated for 3–5 min at 37°C with biotinylated Fab secondary antibodies (goat anti–guinea pig, 4–12 μg/ml; Jackson Immunoresearch) in imaging medium. After washes, cells were incubated for 1 min with streptavidin-coated quantum dots (QDs) emitting at 605 nm (1 nM; Invitrogen) in borate buffer (50 mM) supplemented with sucrose (200 mM) or in PBS (1 M; Invitrogen) supplemented with 10% casein (vol/vol; Sigma-Aldrich). Washing and incubation steps were all done in imaging medium. To assess the membrane dynamics of GABA_A_R α2 subunit at inhibitory synapses in neurons expressing the eGFP-DN mutant, inhibitory synapses were stained by incubating live neurons for 48 h at 37°C in a 5% CO_2_ humidified incubator with a primary VGAT antibody directly coupled to Oyster550 (1:200, Synaptic Systems) diluted in conditioned maintenance medium.

### Single-particle tracking and analysis

Cells were imaged using an Olympus IX71 inverted microscope equipped with a 60× objective (NA 1.42; Olympus) and a Lambda DG-4 monochromator (Sutter Instrument). Individual images of gephyrin-eGFP and homer1c-GFP, and QD real time recordings (integration time of 75 ms over 600 consecutive frames) were acquired with Hamamatsu ImagEM EMCCD camera and MetaView software (Meta Imaging 7.7). Cells were imaged within 45 min after labeling.

QD tracking and trajectory reconstruction were performed with Matlab software (The Mathworks). One to two subregions of dendrites were quantified per cell. In cases of QD crossing, the trajectories were discarded from analysis. Trajectories were considered synaptic when overlapping with the synaptic mask of gephyrin-eGFP or VGAT-Oyster550 clusters, or extrasynaptic for spots 2 pixels (380 nm) away (Lévi et al., 2008). Values of the mean square displacement (MSD) plot versus time were calculated for each trajectory by applying the relation

$$\mathrm{MSD}\left(n\tau \right)=\frac{1}{N-n}\sum_{i=1}^{N-n}\left(\left\{x{\left[\left(i=n\right)\tau -x\left(i\tau \right)\right]}^{2}\right\}+{\left\{y\left[\left(i+n\right)\tau \right]-y\left(i\tau \right)\right\}}^{2}\right),$$

(Saxton and Jacobson, 1997), where τ is the acquisition time, *N* is the total number of frames, and *n* and *i* are positive integers with *n* determining the time increment. Diffusion coefficients (*D*) were calculated by fitting the first four points without origin of the MSD versus time curves with the equation:MSD(nτ)=4Dnτ+b, where *b* is a constant reflecting the spot localization accuracy. Synaptic dwell time was defined as the duration of detection of QDs at synapses on a recording divided by the number of exits as detailed previously (Ehrensperger et al., 2007; Charrier et al., 2010). Dwell times ≤5 frames were not retained. The explored area of each trajectory was defined as the MSD value of the trajectory at two different time intervals of 0.42 and 0.45 s (Renner et al., 2012).

### PALM imaging

Photoactivatable localization microscopy (PALM) imaging on fixed samples was conducted on an inverted N-STORM Nikon Eclipse Ti microscope with a 100× oil-immersion objective (NA 1.49) and an Andor iXon Ultra EMCCD camera (image pixel size, 160 nm), using specific lasers for PALM imaging of Dendra2 (405 and 561 nm). Videos of 10,000 frames were acquired at frame rates of 50 ms. The *z* position was maintained during acquisition by a Nikon perfect focus system. Single-molecule localization and 2D image reconstruction was conducted as described in Specht et al. (2013) by fitting the PSF of spatially separated fluorophores to a 2D Gaussian distribution. The position of fluorophore were corrected by the relative movement of the synaptic cluster by calculating the center of mass of the cluster throughout the acquisition using a partial reconstruction of 2000 frames with a sliding window (Specht et al., 2013). PALM images were rendered by superimposing the coordinates of single-molecule detections, which were represented with 2D Gaussian curves of unitary intensity and SDs representing the localization accuracy (σ = 20 nm). To correct multiple detections coming from the same pDendra2 molecule (Specht et al., 2013), we identified detections occurring in the vicinity of space (2× σ) and time (15 s) as belonging to the same molecule. The surface of gephyrin clusters and the densities of gephyrin molecules per square micrometer were measured in reconstructed 2D images through cluster segmentation based on detection densities. The threshold to define the border was set to 1000 detections/µm^2^, taking into account the reported gephyrin densities in synapses (Specht et al., 2013; Fig. 3*B*). Briefly, all pixels (PALM pixel size = 20 nm) containing <2 detections were considered empty, and their intensity value was set to 0. The intensity of pixels with ≥2 detections was set to 1. The resulting binary image was analyzed with the function “regionprops” of Matlab to extract the surface area of each cluster identified by this function. Density was calculated as the total number of detections in the pixels belonging to a given cluster, divided by the area of the cluster.

### Statistics

Sampling corresponds to the number of quantum dots for SPT, the number of cells for ICC, and the number of synapses for PALM. Sample size selection for experiments was based on published experiments, pilot studies, and in-house expertise. All results were used for analysis except in a few cases. Cells with signs of suffering (apparition of blobs, fragmented neurites) were discarded from the analysis. Means ± SEM are shown, and median values are indicated with their interquartile range (IQR, 25%–75%). Means were compared using the nonparametric Mann–Whitney test (immunocytochemistry, dwell time comparison, PALM quantifications) using SigmaPlot 12.5 (Systat Software). For diffusion coefficient and explored area values having nonnormal distributions, a nonparametric Kolmogorov–Smirnov test was run in Matlab. Differences were considered significant for *p*-values <5%.

## Results

### eGFP-gephyrin mutants exhibit different clustering properties in culture

Signaling pathways that converge onto gephyrin scaffolding properties influence GABA_A_R synaptic transmission. Hence, mimicking phosphorylation/dephosphorylation events that influence gephyrin clustering can help gain critical insights into nanoscale regulation of GABA_A_Rs at synaptic sites. ERK1/2 phosphorylation at the S268 residue results in smaller gephyrin clusters (Tyagarajan et al., 2013); hence, we selected phosphomimetic eGFP-gephyrin-S268E mutant to study the impact of smaller clusters on receptor diffusion. Similarly, pharmacological blockade of the GSK3β pathway or eGFP-gephyrin-S270A mutant expression increases gephyrin cluster number and size (Tyagarajan et al., 2011). We selected the eGFP-S270A mutant to understand how larger clusters would impact receptor diffusion. eGFP-gephyrin dominant negative (DN) mutant in primary neurons not only abolishes gephyrin clustering and reduces surface expression of GABA_A_Rs, but also significantly decreases GABAergic mIPSC amplitude and frequency (Ghosh et al., 2016). Hence, we selected eGFP-DN mutant to evaluate how cluster disruption would impact synaptic anchoring and surface diffusion of GABA_A_Rs.

**Figure 1.**
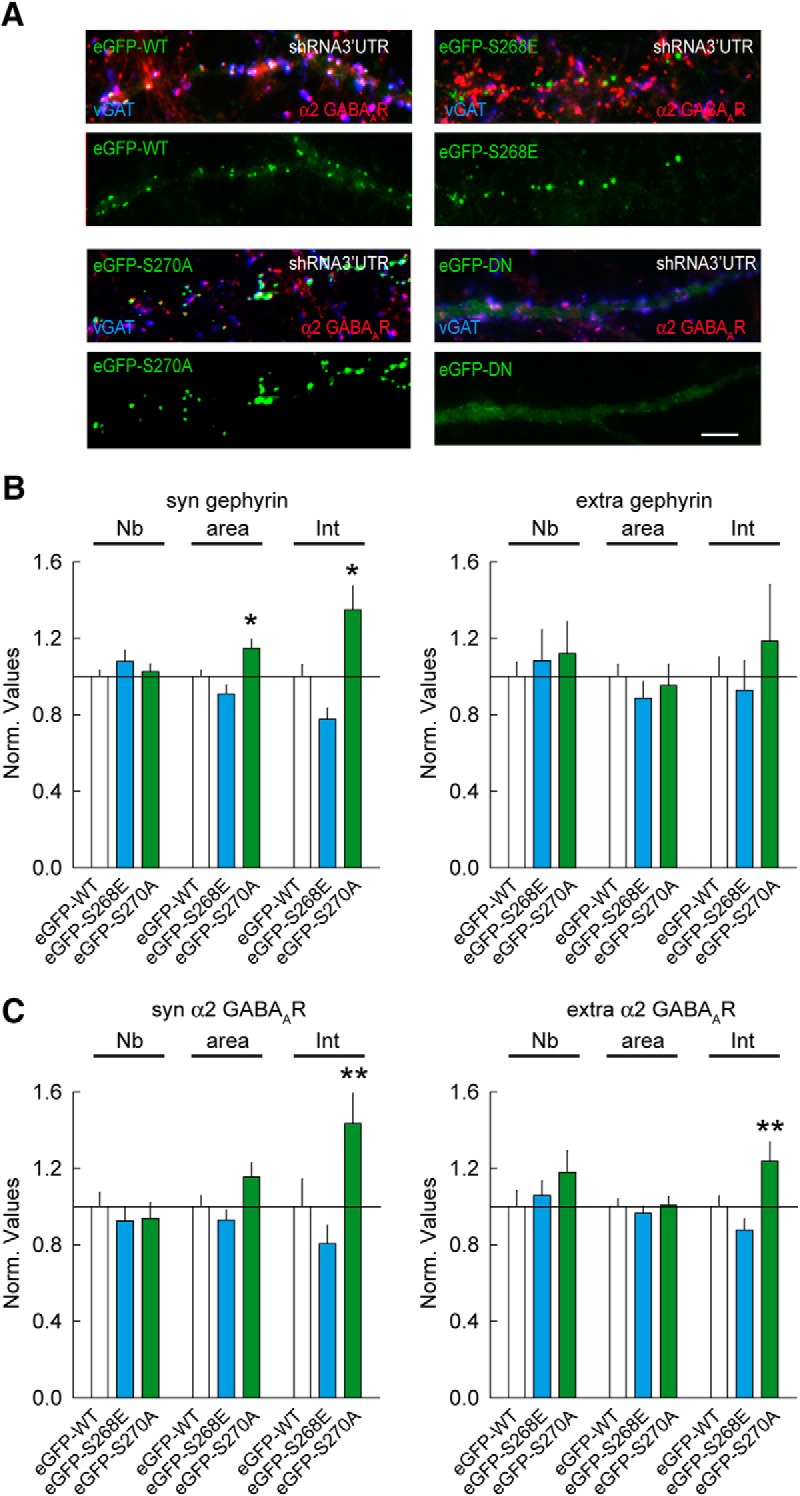
Morphologic characterization of eGFP-gephyrin and its mutant variants. ***A***, Representative images of primary hippocampal neurons cotransfected with eGFP-WT, eGFP-S268E, eGFP-S270A, or eGFP-DN and shRNA-3′UTR. eGFP-gephyrin clusters (green), α2 GABA_A_Rs (red), and VGAT (blue) are shown. Scale bar, 10 μm. ***B***, Quantification of eGFP-gephyrin cluster density, cluster area, and intensity shows larger eGFP-S270A clusters compared with eGFP-WT at synapses. S268E: WT *n* = 66 cells, S268E *n* = 60 cells, 4 cultures. Syn: Cluster Number (Nb) *p* = 0.42, area *p* = 0.22, intensity *p* = 0.05. Extra: Nb *p* = 0.99, Area *p* = 0.66, Intensity *p* = 0.44. S270A: WT *n* = 86 cells, S270A: *n* = 74 cells, 6 cultures. Syn: Nb *p* = 0.77, Area *p* = 0.02, Intensity *p* = 0.02. Extra: Nb *p* = 0.39, Area *p* = 0.42, Intensity *p* = 0.15. ***C***, Quantification for α2 GABA_A_R clusters shows significantly more receptors in eGFP-S270A mutant clusters. S268E: WT *n* = 52 cells, S268E *n* = 47 cells, 3 cultures. Syn: Nb *p* = 0.48, Area *p* = 0.46, Intensity *p* = 0.6. Extra: Nb *p* = 0.46, area *p* = 0.63, intensity *p* = 0.22. S270A: WT *n* = 52 cells, S270A *n* = 39 cells, 3 cultures. Syn: Nb *p* = 0.56, Area *p* = 0.08, Intensity *p* = 0.008. Extra: Nb *p* = 0.008, Area *p* = 0.81, Intensity *p* = 0.29. Data shown as mean ± SEM. Values were normalized to the corresponding control values. Statistics *, *p* ≤ 0.05, **, *p* ≤ 0.01 (Mann–Whitney rank sum test).

Primary hippocampal neurons were cotransfected at 14 DIV with eGFP-gephyrin WT (eGFP-WT), eGFP-S268E, eGFP-S270A, or eGFP-DN along with shRNA targeting the gephyrin 3′ UTR (to minimize the influence of endogenous gephyrin expression on mutant phenotypes). Before studying the influence of altered gephyrin clustering on GABA_A_R diffusion properties, we confirmed the respective gephyrin mutant morphology 6–9 d after transfection. Representative images of neurons expressing either eGFP-WT or eGFP-S268E, eGFP-S270A, eGFP-DN variants are shown (Fig. 1*A*). We stained for the α2 GABA_A_R subunit to study the relation of eGFP-gephyrin with receptors. Quantification for eGFP-gephyrin cluster density (Nb), cluster size (area), and intensity (Int) showed a tendency for reduced clustering for the S268E mutant and increased clustering for the S270A mutant (Fig 1*B*). The impact of the gephyrin S270A mutation on gephyrin cluster area and intensity was more pronounced, in comparison to the S268E mutant. As expected, eGFP-DN failed to cluster (data not shown). Similar to the observed changes in eGFP-gephyrin morphology, quantification of cluster intensity for α2 GABA_A_R showed a significant increase in neurons expressing eGFP-S270A, whereas eGFP-S268E expressing neurons showed only a modest reduction in α2 (Fig. 1*C*). The neurons expressing eGFP-DN showed very little α2 GABA_A_R staining (data not shown).

### Influence of eGFP-gephyrin mutants on GABA_A_R surface diffusion

**Figure 2.**
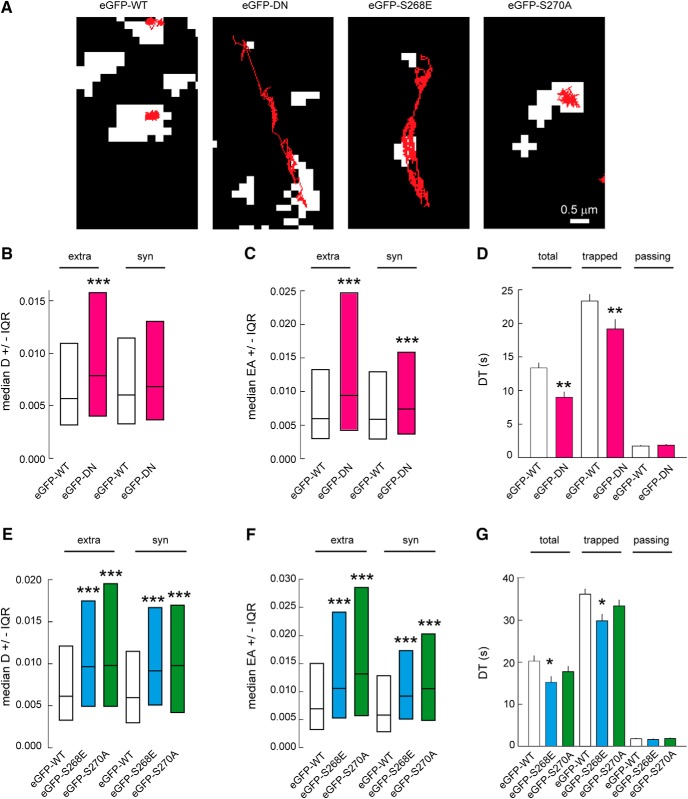
Membrane dynamics of α2 GABA_A_R is influenced by gephyrin phosphorylation. ***A***, Example traces of QD trajectories (red) overlaid with fluorescent synaptic clusters (white) of VGAT-Oyster550 for eGFP-DN transfected neurons or with eGFP-gephyrin clusters for eGFP-WT, eGFP-S268E, or eGFP-S270A expressing cells. Scale bar, 0.5 µm. ***B***, Median diffusion coefficients D of α2 GABA_A_R in neurons transfected with either eGFP-WT or eGFP-DN. Extra: WT *n* = 975 QDs, DN *n* = 491 QDs, *p* = 4.5 × 10^−34^; Syn: WT *n* = 306 QDs, DN *n* = 173 QDs, *p* = 0.36. ***C***, Quantification of explored area EA of α2 GABA_A_R, Extra: WT *n* = 2925 QDs, DN *n* = 1473 QDs, *p* = 3.8 × 10^−23^; Syn: WT *n* = 918 QDs, DN *n* = 519 QDs, *p* = 4.4 × 10^−4^. ***D***, Dwell time DT of α2 GABA_A_R at synapses in neurons transfected with either eGFP-WT or eGFP-DN. Quantification of all QDs (total), trapped (DT < 5.9 s), and passing (DT > 5.9 s) QDs at inhibitory synapses. Significant decrease in synaptic dwell time for total and trapped QDs was observed but not for passing ones. Total: WT *n* = 436 QDs, DN *n* = 262 QDs, *p* = 0.001; Trapped: WT *n* = 235 QDs, DN *n* = 108 QDs, *p* = 8.0 × 10^−3^; Passing: WT *n* = 201 QDs, DN *n* = 154 QDs, *p* = 0.19. ***E***, Quantification of diffusion coefficients of α2 GABA_A_R showing increased receptor mobility at extrasynaptic (extra) and synaptic (syn) sites in neurons transfected with eGFP-S268E or eGFP-S270A, compared with eGFP-WT expressing cells. Extra: WT *n* = 1820 QDs, S268E *n* = 1273 QDs, *p* = 1.1 × 10^−22^, S270A *n* = 1658, *p* = 2.9 × 10^−27^. Syn: WT *n* = 461 QDs, S268E *n* = 326 QDs, *p* = 2.4 × 10^−8^, S270A *n* = 340, *p* = 1.8 × 10^−8^. ***F***, Quantification of α2 GABA_A_R explored area EA, Extra: WT *n* = 5460 QDs, S268E *n* = 3807 QDs, *p* = 6.8 × 10^−52^, S270A *n* = 5355, *p* = 2.2 × 10^−101^. Syn: WT *n* = 1383 QDs, S268E *n* = 978 QDs, *p* = 7.4 × 10^−23^, S270A *n* = 2208, *p* = 1.2 × 10^−33^. ***G***, Quantification of α2 GABA_A_R dwell time DT in neurons expressing eGFP-WT, eGFP-S268E, or eGFP-S270A. Calculations were done for all QDs (total), (trapped), or (passing) QDs at inhibitory synapses. Decrease in dwell time for the whole or trapped population of QDs was seen in synapses expressing eGFP-S268E but not in synapses containing eGFP-S270A. Total: WT *n* = 251 QDs, S268E *n* = 176 QDs, *p* = 0.013, S270A *n* = 216 QDs, *p* = 0.31; Trapped: WT *n* = 135 QDs, S268E *n* = 85 QDs, *p* = 0.002, S270A *n* = 109 QDs, *p* = 0.28; Passing: WT *n* = 116 QDs, S268E *n* = 91 QDs, *p* = 0.24, S270A *n* = 107 QDs, *p* = 0.98. All data are from six independent experiments. In ***B***, ***C***, ***E***, and ***F***, data are presented as median values ± 25%–75% IQR, ***, *p* ≤ 0.001 (Kolmogorov–Smirnov test). In ***D*** and ***G***, data are presented as mean ± SEM. *, *p* ≤ 0.05, **, *p* ≤ 0.01 (Mann–Whitney rank sum test). D in µm^2^/s, EA in µm^2^, DT in s.

GABA_A_Rs are known to exhibit faster mobility at extrasynaptic sites compared with synaptic sites. Because of their interaction with the main scaffolding molecule gephyrin, GABA_A_Rs are slowed down and confined at synapses. This diffusion-capture of GABA_A_Rs is modulated by neuronal activity and constitutes an important basis for synaptic plasticity (Petrini and Barberis, 2014). The expression of specific eGFP-gephyrin mutations allows us to lock the scaffold into different conformations and study its influence on GABA_A_R surface diffusion. To achieve this, we assessed the lateral mobility of α2 GABA_A_R using quantum dot–based single-particle tracking (QD-SPT). Live imaging over 600 constitutive frames at 75 Hz was used to record individual trajectories, and the trajectories were later analyzed using custom software (Fig. 2*A*; see Methods). As a proof of concept, we first tested the effect of total gephyrin cluster removal on α2 GABA_A_R surface dynamics by expressing the eGFP-DN mutant. However, given that eGFP-DN has a diffuse expression, to distinguish synaptic and extrasynaptic α2 clusters we preloaded presynaptic GABAergic terminals using VGAT-Oyster550 antibody. The expression of the eGFP-DN mutant increased the surface exploration of QDs at both extrasynaptic and synaptic sites compared with control eGFP-WT. Quantification of the α2 GABA_A_R diffusion coefficient showed a 1.4-fold increase for extrasynaptic receptors and a 1.2-fold increase for synaptic receptors in eGFP-DN–expressing neurons (Fig. 2*B*). Areas explored by α2 GABA_A_Rs also showed a 1.6-fold increase at extrasynaptic sites and a 1.3-fold increase at synaptic sites in eGFP-DN–expressing neurons (Fig. 2*C*). These observations support the notion that gephyrin slows down and confines GABA_A_Rs not only at synapses but also at extrasynaptic sites (Ehrensperger et al., 2007).

Synaptic dwell time values can be discriminated from “trapped” receptors (dwell time >5.9 s) and “passing” receptors (dwell time ≤5.9 s; Renner et al., 2012). Quantification of α2 GABA_A_R dwell time confirmed a 1.3-fold faster escape time of receptors in neurons expressing the eGFP-DN mutant (Fig. 2*D*). We did not observe any difference in this rate for passing receptors. This is an indication that the observed reduction of trapped receptors is not due to increased membrane viscosity, but rather to gephyrin scaffold’s influence on GABA_A_R surface mobility. Thus, we concluded that the diffuse DN gephyrin relieved GABA_A_R α2 diffusion constraints, leading to synaptic escape of receptors.

If gephyrin clustering can indeed influence receptor diffusion, then S268E and S270A modifications must have an influence on α2 GABA_A_R surface mobility. To test this, we transfected the eGFP-S268E or eGFP-S270A mutants and measured surface mobility at extrasynaptic and synaptic locations. Superimposition of trajectories with fluorescent images of eGFP-gephyrin allowed us to distinguish synaptic versus extrasynaptic α2 GABA_A_Rs. Neurons transfected with eGFP-S268E exhibited an increase in surface exploration of individual trajectories (Fig. 2*A*). This was consistent with the observed increase in diffusion coefficients at both extrasynaptic and synaptic sites (Fig. 2*E*). Similarly, quantification of explored areas at both extrasynaptic and synaptic sites showed significant increases (Fig. 2*F*). If reducing gephyrin cluster size facilitates α2 diffusion, then we would expect shorter dwell time at synaptic sites. Indeed, we report reduced dwell time for trapped α2 GABA_A_Rs in eGFP-S268E–transfected neurons (Fig. 2*G*). Therefore the use of eGFP-S268E gephyrin mutant shows that the reduction in gephyrin cluster size causes an increase in GABA_A_R diffusion, while reducing synaptic dwell time.

On the other hand, in eGFP-S270A–transfected neurons, the α2 GABA_A_Rs showed increased surface exploration of individual trajectories at synapses (Fig. 2*A*). Unexpectedly, diffusion coefficients and surface exploration of α2 extrasynaptic and synaptic GABA_A_Rs were significantly increased in eGFP-S270A–transfected neurons (Fig. 2*E*,*F*). However, analysis showed no reduction in α2 GABA_A_R dwell time at synaptic sites (Fig. 2*G*). We thus concluded that the increase in receptor mobility at synapses in S270A-transfected neurons does not correlate with what we may expect from a larger scaffold, suggesting additional regulations are at play.

### Superresolution PALM microscopy reveals differential packing of gephyrin scaffold

We turned to quantitative nanoscopic imaging to understand the influence of phosphorylation on gephyrin scaffold organization. Using photoactivated localization microscopy (PALM), we estimated localization accuracy from several detections of the same fluorophore from subsequent image frames (Specht et al., 2013). The spatial resolution of PALM is within the range of ∼25–30 nm; hence, image segmentation of the rendered PALM images can resolve substructure organization within a gephyrin cluster that are not discernable using diffraction-limited imaging (Specht et al., 2013).

**Figure 3.**
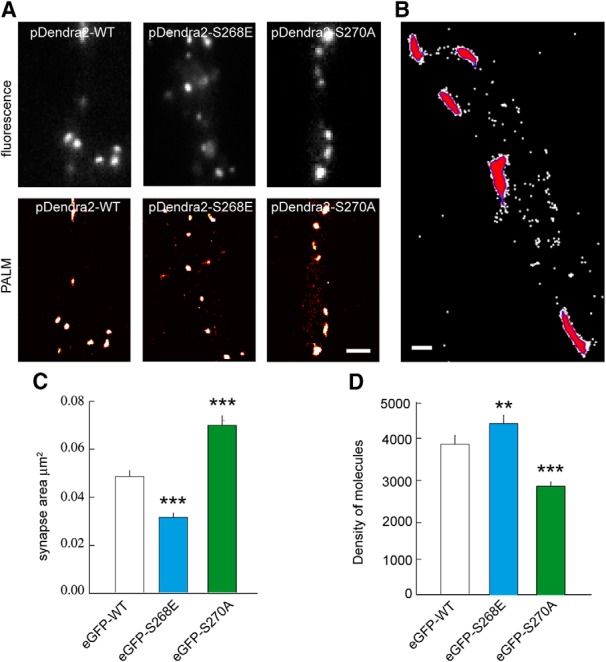
PALM imaging showing gephyrin phosphorylation influences scaffold packing. ***A***, Epifluorescence (top) and PALM (bottom) imaging of the same dendritic regions in neurons expressing pDendra2-WT, -S268E, or -S270A mutant. Scale bar, 1 µm. ***B***, Representative image of cluster segmentation (red) based on local density of molecules detected (white dots) using a threshold of 1000 detections/µm^2^ (blue). Scale bar, 200 nm. ***C***, Quantification of eGFP cluster area using PALM shows reduction in cluster size for eGFP-S268E and increase in cluster size for eGFP-S270A compared with eGFP-WT. WT *n* = 313 synapses, S268E *n* = 277 synapses, S270A *n* = 290 synapses, *p* < 0.001, 4 cultures. ***D***, Quantification of density of gephyrin molecules per square micrometer using PALM in transfected neurons. Neurons expressing eGFP-S268E exhibit denser gephyrin packing, and neurons expressing eGFP-S270A exhibit less dense packing of gephyrin compared with eGFP-WT. Data are presented as mean ± SEM. **, *p* = 0.006; ***, *p* ≤ 0.001 (Mann–Whitney rank sum test).

Employing fluorescence imaging on primary hippocampal neurons cotransfected with photoconvertible pDendra2-WT, pDendra2-S268E, or pDendra2-S270A and shRNA 3′ UTR showed a clustering phenotype consistent with eGFP-gephyrin and its mutant variants (Fig. 3*A*). PALM image cluster segmentation was established based on local density of detections using a threshold of 1000 detections/μm^2^ (Fig. 3*B*). Image segmentation allows us to estimate the mean surface area of a given pDendra2-WT cluster. In this case, quantification showed pDendra2-WT clusters to be 0.054 ± 0.003 μm^2^, corresponding to the mean diameter of 262 nm as has been reported earlier (Specht et al., 2013). pDendra2-S268E quantifications showed a significant reduction in mean surface area to 0.035 ± 0.002 μm^2^, and consistent with our expectations, pDendra2-S270A showed an increase in cluster area of 0.078 ± 0.005 μm^2^ (Fig. 3*C*).

We next tried to correlate the estimated size of gephyrin clusters to their respective densities. Our analysis showed 3919.7 ± 227.9 molecules/μm^2^ of pDendra2-WT within a cluster (Fig. 3*D*). pDendra2-S268E showed a significantly increased molecular density (4457.5 ± 221.6) despite having a smaller cluster area. In contrast, pDendra2-S270A mutant shows a significantly reduced molecular density (2819.8 ± 117.6), despite having a larger surface area (Fig. 3*D*).

Our data indicate that there is no correlation between the diffusion properties of GABA_A_Rs despite the relative size difference between S268E and S270A gephyrin clusters. However, there is a strong correlation between gephyrin phosphorylation and cluster microdomain compaction. The compaction of the scaffold or the increased spacing between gephyrin molecules may perturb the organization of the gephyrin microdomain, thereby altering gephyrin-receptor binding properties. We cannot exclude the possibility that the mutations directly affect receptor-binding properties independently of their effect on the mesh.

### Prolonged neuronal activity influences gephyrin and GABA_A_R clustering as well as GABA_A_R diffusion

**Figure 4.**
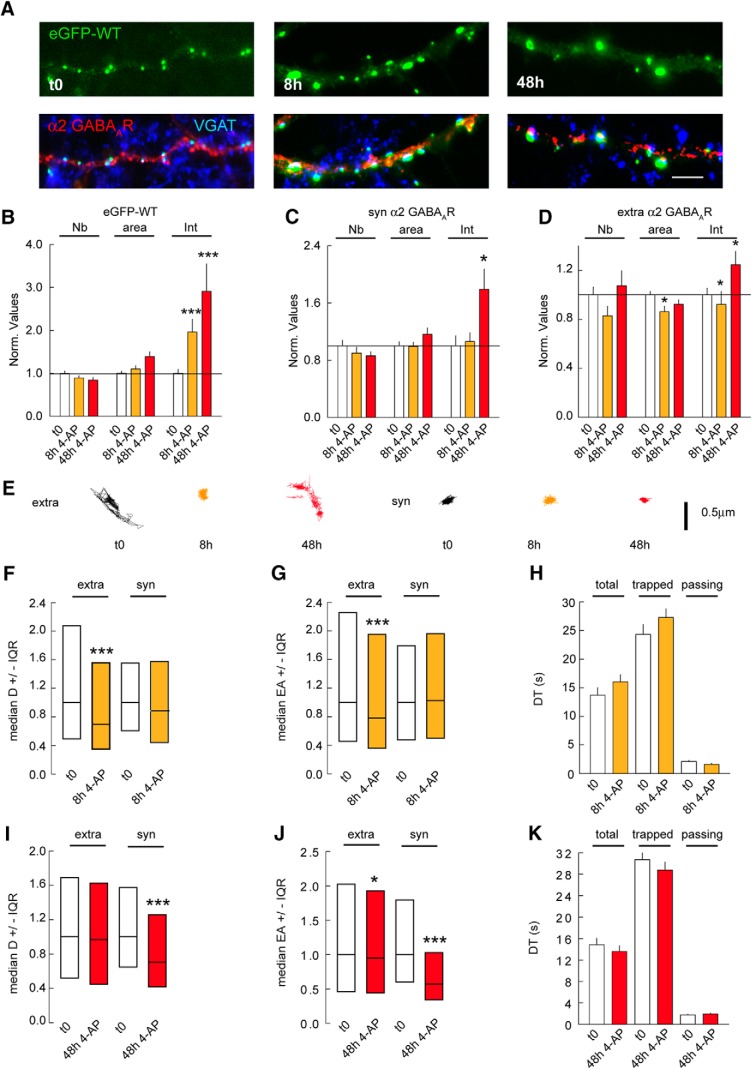
Gephyrin clustering influences GABA_A_R lateral diffusion. ***A***, Morphology of eGFP-WT (green) after 8 and 48 h of 4-AP application; VGAT (blue), GABA_A_R α2 (red) at 21 DIV. Scale bar, 10 µm. ***B***, Quantification of eGFP-WT clusters after 8 and 48 h of 4-AP application. t0 *n* = 55 cells, 8 h *n* = 46 cells, 48 h *n* = 55 cells, 3 cultures. Cluster Nb: 0–8 h: *p* = 0.13, 0–48 h: *p* = 0.002; Area: 0–8 h: *p* = 0.5, 0–48 h: *p* = 0.001; Intensity: 0–8 h: *p* < 0.001, 0–48 h: *p* < 0.001. ***C***, Quantification of synaptic α2 GABA_A_R clusters after 8 and 48 h of 4-AP compared with mock treated control. t0 *n* = 52 cells, 8 h *n* = 43 cells, 48 h *n* = 53 cells, 3 cultures. Cluster Nb: 0–8 h: *p* = 0.4, 0–48 h: *p* = 0.3; Area: 0–8 h: *p* = 0.8, 0–48 h: *p* = 0.8; Intensity: 0–8 h: *p* = 0.5, 0–48 h: *p* = 0.03. ***D***, Quantification of extrasynaptic α2 GABA_A_R clusters after 8 and 48 h of 4-AP compared with mock treated control. t0 *n* = 52 cells, 8 h *n* = 43 cells, 48 h *n* = 53 cells, 3 cultures. Cluster Nb: 0–8 h: *p* = 0.2, 0–48 h: *p* = 0.9; Area: 0–8 h: *p* = 0.02, 0–48 h: *p* = 0.3; Intensity: 0–8 h: *p* = 0.05, 0–48 h: *p* = 0.022. ***E***, Example trace of α2 GABA_A_R trajectories showing surface exploration of extrasynaptic and synaptic receptors after 8 and 48 h of 4-AP exposure. Scale bar, 0.5 µm. ***F***, Quantification of diffusion coefficients of α2 GABA_A_R after 8 h of 4-AP exposure. Extra; t0 *n* = 450 QDs, WT 4AP 8 h *n* = 961 QDs, *p* = 1.96 10^−7^. Syn; t0 *n* = 103 QDs, 8 h *n* = 138 QDs, *p* = 0.22; 2 cultures. ***G***, Quantification of explored area EA of α2 GABA_A_R after 8 h of 4-AP application. Extra; t0 *n* = 1347 QDs, 8 h *n* = 5265 QDs, *p* = 6.4 × 10^−9^. Syn; t0 *n* = 308 QDs, 8 h *n* = 708 QDs, *p* = 0.63. ***H***, Quantification of synaptic dwell time DT of α2 GABA_A_R showing no impact after 8 h of 4-AP for total, trapped, or passing receptor population. Total: t0 *n* = 151 QDs, 8 h *n* = 206 QDs, *p* = 0.073; Trapped: t0 *n* = 80 QDs, 8 h *n* = 116 QDs, *p* = 0.36; Passing: t0 *n* = 78 QDs, 8 h *n* = 90 QDs, *p* = 0.02. ***I***, Quantification of diffusion coefficients of α2 GABA_A_R after 48 h of 4-AP application. Extra: t0 *n* = 777 QDs, 48 h *n* = 174 QDs, *p* = 0.69. Syn: t0 *n* = 126 QDs, 48 h *n* = 213 QDs, *p* = 1.4 × 10^−4^. ***J***, Quantification of explored area EA of α2 GABA_A_R after 48 h of 4-AP application. Extra: t0 *n* = 2331 QDs, 48 h *n* = 5508 QDs, *p* = 0.045. Syn: t0 *n* = 378 QDs, 48 h *n* = 717 QDs, *p* = 2.2 × 10^−20^. ***K***, Quantification of α2 GABA_A_R dwell time after 48 h of 4-AP application. Total: t0 *n* = 201 QDs, 48 h *n* = 254 QDs, *p* = 0.74. Trapped: t0 *n* = 91 QDs, 48 h *n* = 110 QDs, *p* = 0.99. Passing: t0 *n* = 110 QDs, 48 h *n* = 144 QDs, *p* = 0.81. In ***B–D***, ***H***, and ***K***, data are presented as mean ± SEM, *, *p* ≤ 0.05; ***, *p* ≤ 0.001 (Mann–Whitney rank sum test). In ***F***, ***G***, ***I***, and ***J***, data are presented as median values ± 25%–75% IQR, *, *p* ≤ 0.05; ***, *p* ≤ 0.001 (Kolmogorov–Smirnov test). In ***B–G***, ***I***, and ***J***, values were normalized to the corresponding control values. In ***H*** and ***K***, DT in s.

Activity-dependent regulation of receptor lateral diffusion is an essential contributor to synapse adaptation (Lüscher et al., 2011). This phenomenon has been explored within the experimental paradigm of short-term (1- to 60-min) drug applications (Bannai et al., 2009, 2015; Muir et al., 2010; Niwa et al., 2012; Petrini et al., 2014). There is accumulating evidence that synaptic adaptations at GABAergic synapses also occur in response to prolonged changes in activity (Rannals and Kapur, 2011; Vlachos et al., 2013; Flores et al., 2015). Hence, we examined whether gephyrin phosphorylation regulates activity-dependent membrane diffusion and synaptic recruitment of α2 GABA_A_Rs. To test this hypothesis, we chronically elevated synaptic activity by treating our primary hippocampal neurons with the potassium channel blocker 4-aminopyridine (4-AP; 100 μM; Chamma et al., 2013) for 8 or 48 h. We used immunocytochemistry to determine the impact of a prolonged activity increase on gephyrin and α2 GABA_A_R clustering (Fig. 4*A*). Quantification across independent experiments showed that fluorescence intensity of eGFP-WT gephyrin clusters increased by 1.95-fold after 8 h and 2.3-fold after 48 h of 4-AP treatment (Fig. 4*B*). Quantification for α2 GABA_A_R cluster intensity after 8 h of 4-AP–induced neuronal activity did not show an increase in receptor accumulation at synapses; however, after 48 h of 4-AP treatment, we found a 1.7-fold increase in receptor density at synaptic sites (Fig. 4*C*). Thus, gephyrin recruitment at synapses precedes that of the receptor in response to chronic changes in activity. In contrast to synaptic clusters, extrasynaptic α2 clusters decreased in size and intensity after 8 h of 4-AP application (Fig. 4*D*). This transient decrease in extrasynaptic α2 clusters intensity is reversed after 48 h of 4-AP, similar to synaptic receptor clusters (Fig. 4*C*,*D*). Therefore, a chronic increase in activity regulates both extrasynaptic and synaptic receptor clustering.

It has been reported that acute 4-AP treatment increases GABA_A_R mobility between synaptic and extrasynaptic sites (Bannai et al., 2009). Hence, we analyzed α2 GABA_A_R surface diffusion at extrasynaptic and synaptic sites after either 8 or 48 h of 4-AP treatment using QD-SPT (Fig. 4*E*). Quantification of the receptor diffusion coefficient showed a 1.3-fold reduction for extrasynaptic receptors; however, the synaptic receptors were not influenced by 8 h of 4-AP treatment (Fig. 4*F*). Consistently, 8 h of 4-AP treatment reduced the explored area for only the extrasynaptic receptors by 1.2-fold (Fig. 4*G*). The receptor dwell time at synaptic sites was also unchanged after 8 h of activity change (Fig. 4*H*). This is consistent with a lack of receptor accumulation at synapses after 8 h of 4-AP treatment.

Contrary to the 8-h 4-AP treatment, 48-h treatment significantly reduced the diffusion coefficients of synaptic α2 receptors by 1.3-fold, while having no effect on the extrasynaptic receptors (Fig. 4*I*). We also observed a 1.3-fold reduction in explored area for synaptic α2 GABA_A_Rs, with only a modest reduction for extrasynaptic receptors (Fig. 4*J*). Unexpectedly, the reduction in the diffusion rate and explored area of synaptic α2 receptors had no influence on the dwell time at synaptic sites (Fig. 4*K*). Therefore, pools of extrasynaptic and synaptic receptor are regulated independently of each other over prolonged activity change.

Altogether, our data show that GABA_A_R lateral diffusion can be regulated on a time scale of days. We observe a decrease in synaptic GABA_A_R diffusion at the 48-h time point and not at 8 h, which is in direct correlation to cluster intensity change observed after 48 h. Therefore, regulation of GABA_A_R diffusion capture accounts for the change in receptor density at synapses on chronic changes in activity.

### PKA and CaMKIIα pathways regulate synaptic scaling at GABAergic postsynaptic sites through gephyrin phosphorylation

**Figure 5.**
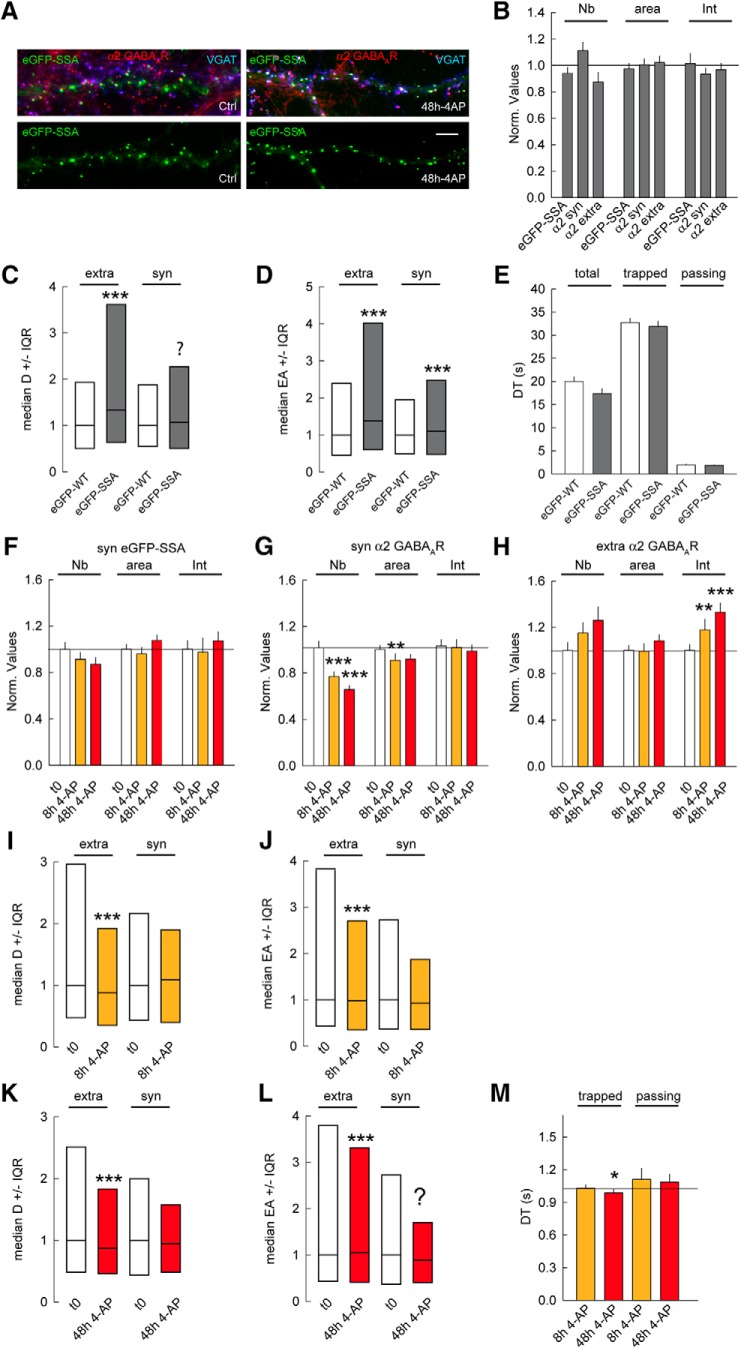
PKA and CaMKIIα signaling pathways regulate gephyrin clustering and α2 GABA_A_R membrane dynamics in conditions of chronic changes of activity. ***A***, Morphologic analysis of neurons transfected with eGFP-S303A/S305A (eGFP-SSA) gephyrin double mutant insensitive to PKA and CaMKIIα signaling pathways. Double staining of VGAT (blue) and α2 GABA_A_R (red) at 21 DIV under control condition (t0) or in the presence of 4-AP for 48 h. Scale bar, 10 µm. ***B***, Quantifications of synaptic eGFP-SSA clusters and synaptic (α2 syn) and extrasynaptic (α2 extra) α2 GABA_A_R clusters in relation to eGFP-WT show minor impact of eGFP-SSA under control condition. eGFP-WT: *n* = 89 cells, eGFP-SSA *n* = 95 cells, 6 cultures. eGFP-SSA: Cluster Nb: *p* = 0.3; Area: *p* = 0.9; Intensity: *p* = 0.5. α2 syn: Cluster Nb: *p* = 0.4; Area: *p* = 0.5; Intensity: *p* = 0.8. α2 extra: Cluster Nb: *p* = 0.2; Area: *p* = 0.4; Intensity: *p* = 0.2. ***C***, Quantification of median diffusion coefficient D of α2 GABA_A_R in neurons expressing eGFP-WT or eGFP-SSA under control condition. Extra: WT *n* = 1166 QDs, SSA *n* = 989 QDs, *p* = 1.5 × 10^−12^; Syn: WT *n* = 312 QDs, SSA *n* = 245 QDs, *p* = 0.08; 4 cultures. ***D***, Quantification of median explored area EA of α2 GABA_A_R in neurons expressing eGFP-WT or eGFP-SSA under control condition. Extra: WT *n* = 3510 QDs, SSA *n* = 2778 QDs, *p* = 3.9 × 10^−18^; Syn: WT *n* = 932 QDs, SSA *n* = 735 QDs, *p* = 3.1 × 10^−4^. ***E***, Quantification of α2 GABA_A_R dwell time DT at synaptic sites in neurons expressing either eGFP-WT or eGFP-SSA. Calculations were done for all QDs (total), (trapped), or (passing) QDs at inhibitory synapses. No significant differences were found between eGFP-WT and eGFP-SSA. Total: WT *n* = 390 QDs, SSA *n* = 335 QDs, *p* = 0.2; Trapped: WT *n* = 229 QDs, SSA *n* = 173 QDs, *p* = 0.4; Passing: WT *n* = 161 QDs, SSA *n* = 162 QDs, *p* = 0.9. ***F***, Quantification of eGFP-SSA clusters after 8 and 48 h of 4-AP application. t0 *n* = 61 cells, 8 h *n* = 52 cells, 48 h *n* = 93 cells, 3–6 cultures. Cluster Nb: 0–8 h: *p* = 0.2, 0–48 h: *p* < 0.001; Area: 0–8 h: *p* = 0.8, 0–48 h: *p* = 0.3; intensity: 0–8 h: *p* = 0.8, 0–48 h: *p* = 0.2. ***G***, Quantification of synaptic α2 GABA_A_R clusters after 8 and 48 h of 4-AP compared with mock treated control. t0 *n* = 53 cells, 8 h *n* = 50 cells, 48 h *n* = 69 cells, 3–6 cultures. Cluster Nb: 0–8 h: *p* < 0.001, 0–48 h: *p* < 0.001; Area: 0–8 h: *p* = 0.002, 0–48 h: *p* = 0.09; Intensity: 0–8 h: *p* = 0.5, 0–48 h: *p* = 0.5. ***H***, Quantification of extrasynaptic α2 GABA_A_R clusters after 8 and 48 h of 4-AP compared with mock treated control. Cluster Nb: 0–8 h: *p* = 0.2, 0–48 h: *p* = 0.1; Area: 0–8 h: *p* = 0.01, 0–48 h: *p* = 0.9; Intensity: 0–8 h: *p* = 0.002, 0–48 h: *p* < 0.001. ***I***, Quantification of α2 GABA_A_R diffusion coefficients in eGFP-SSA expressing cells after 8 h of 4-AP exposure. Extra: t0 *n* = 787 QDs, 4AP 8 h *n* = 365 QDs, *p* = 3.6 × 10^−4^. Syn: t0 *n* = 212 QDs, 8 h *n* = 187 QDs, *p* = 0.4; 5 cultures. ***J***, Quantification of explored area EA of α2 GABA_A_R after 8 h of 4-AP application. Extra: t0 *n* = 1869 QDs, 8 h *n* = 1092 QDs, *p* = 0.002. Syn: t0 *n* = 753 QDs, 8 h *n* = 558 QDs, *p* = 0.09. ***K***, Quantification of α2 GABA_A_R diffusion coefficients in eGFP-SSA expressing cells after 48 h of 4-AP exposure. Extra: t0 *n* = 1098 QDs, 4AP 48 h *n* = 734 QDs, *p* = 0.002. Syn: t0 *n* = 287 QDs, 48 h *n* = 198 QDs, *p* = 0.2; 5 cultures. ***L***, Quantification of explored area EA of α2 GABA_A_R after 48 h of 4-AP application. Extra: t0 *n* = 2169 QDs, 48 h *n* = 1500 QDs, *p* = 0.04. Syn; t0 *n* = 633 QDs, 48 h *n* = 510 QDs, *p* = 0.002. ***M***, Quantification of α2 GABA_A_R dwell time DT in neurons expressing eGFP-SSA after 8 or 48 h of 4-AP application. Calculations were done for trapped or passing QDs at inhibitory synapses. Trapped: 8 h: *n* = 189 QDs, *p* = 0.3; 48 h: *n* = 166 QDs, *p* = 0.1; Passing: 8 h: *n* = 76 QDs, *p* = 0.3; 48 h: *n* = 132 QDs, *p* = 0.9. In ***B***, ***E***, ***F–H***, and ***M***, data are presented as mean ± SEM. **, *p* < 0.01; ***, *p* ≤ 0.001 (Mann–Whitney rank sum test). In ***C***, ***D***, ***I***, and ***L***, data are presented as median values ± 25%–75% IQR; ***, *p* ≤ 0.001 (Kolmogorov–Smirnov test). In all graphs except ***E***, values were normalized to the corresponding control values.

To identify signaling cascades that couple the gephyrin scaffold to GABA_A_Rs for activity-dependent synaptic recruitment, we focused on the protein kinase A (PKA) and CaMKIIα pathways. NMDA receptor–dependent compensatory adaptations at the GABAergic postsynaptic sites have been reported to be facilitated by gephyrin phosphorylation at PKA and CaMKIIα locations (Flores et al., 2015). We thus transfected the eGFP-S303A/S305A (SSA) mutant (insensitive to PKA- and CaMKIIα-dependent phosphorylation) into our primary hippocampal neurons and treated the neurons for 8 or 48 h with 4-AP. We did not observe differences between eGFP-WT and eGFP-SSA cluster number, cluster size, and fluorescence intensity in control conditions (Fig. 5*A*,*B*). Similarly, the SSA mutant did not significantly influence the synaptic or extrasynaptic clustering of α2 GABA_A_Rs (Fig. 5*B*). In contrast, the SSA mutation increased the diffusion coefficient and explored area of α2 GABA_A_Rs at both extrasynaptic and synaptic sites (Fig. 5*C*,*D*). This increase in receptor mobility did not correlate with what we expected from a normal-size scaffold. However, the α2 GABA_A_Rs dwell time at inhibitory synapses did not differ between eGFP-SSA– and eGFP-WT–transfected neurons (Fig. 5*E*), indicating that the increase in receptor mobility was not accompanied by a faster synaptic escape of receptors. This is consistent with a lack of effect of the SSA mutant on α2 GABA_A_Rs clustering at synapses.

The expression of the eGFP-SSA mutant was sufficient to prevent the 4-AP (8- or 48-h) induced gephyrin and α2 GABA_A_Rs cluster growth at synapses (Fig. 5*A*,*F*,*G*). Interestingly, 8 and 48 h after 4-AP application, extrasynaptic α2 cluster intensity increased in eGFP-SSA–transfected neurons (Fig. 5*H*). This indicated that receptor clustering at extrasynaptic sites at the 8-h treatment time point is dependent on PKA and CaMKIIα phosphorylation. However, at 48 h, receptor accumulation is independent of these two pathways. Hence, an additional pathway permits GABA_A_R recruitment, in particular at extrasynaptic sites, after chronic changes in activity.

We also analyzed the effect of the SSA mutant on α2 GABA_A_Rs surface diffusion. Similar to wild-type gephyrin, SSA reduced diffusion coefficient and surface exploration of α2 GABA_A_Rs at extrasynaptic sites after 8 h of 4-AP (Fig. 5*I*,*J*). This effect was maintained also after 48-h treatment (Fig. 5*K*,*L*). In contrast to wild-type gephyrin, SSA mutant increased α2 GABA_A_R confinement and decreased dwell time of GABA_A_Rs at synapses after 48 h of 4-AP (Fig. 5*K–M*). The passing α2 GABA_A_Rs remained unchanged at synapses after 8 or 48 h of 4-AP (Fig. 5*M*). Hence, our results indicate that gephyrin scaffold reorganization via PKA- and CaMKIIα-dependent phosphorylation at S303 and S305 is essential for GABA_A_R diffusion at synapses but not at extrasynaptic sites in response to chronic changes in activity.

### Synapse scaling is independent of the ERK1/2 pathway

**Figure 6.**
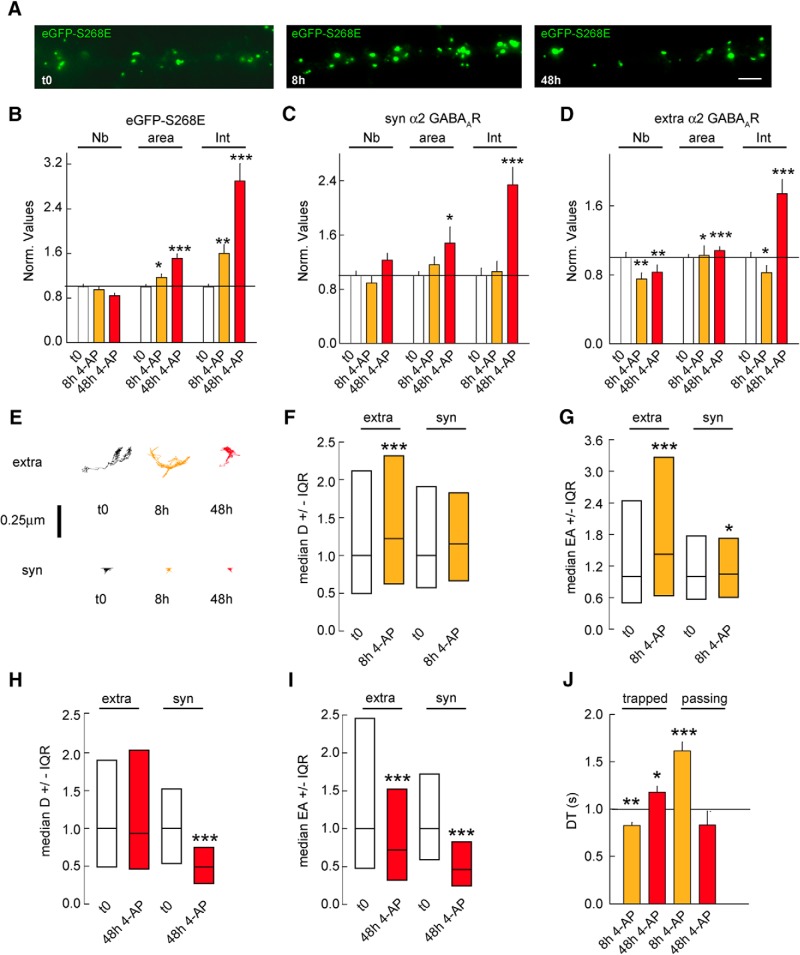
The ERK1/2 pathway does not influence structural synaptic adaptation. ***A***, Morphologic analysis of eGFP-S268E in control (t0) or after 4-AP application for 8 or 48 h. Scale bar, 10 µm. ***B***, Quantification of eGFP-S268E clusters after 8 or 48 h of 4-AP application. t0 *n* = 50 cells, 8 h *n* = 54 cells, 48 h *n* = 55 cells, 3 cultures. Cluster Nb: 0–8 h: *p* = 0.2, 0–48 h: *p* = 0.004; Area: 0–8 h: *p* = 0.02, 0–48 h: *p* < 0.001; Intensity: 0–8 h: *p* = 0.003, 0–48 h: *p* < 0.001. 3 cultures. ***C***, Quantification of synaptic α2 GABA_A_R clusters after 8 h and 48 h of 4-AP compared with mock treated control. t0 *n* = 47 cells, 8 h *n* = 50 cells, 48 h *n* = 62 cells, 3-4 cultures. Cluster Nb: 0–8 h: *p* = 0.08, 0–48 h: *p* = 0.5; Area: 0–8 h: *p* = 0.8, 0–48 h: *p* = 0.03; Intensity: 0–8 h: *p* = 0.5, 0–48 h: *p* < 0.001. ***D***, Quantification of extrasynaptic α2 GABA_A_R clusters after 8 and 48 h of 4-AP compared with mock treated control. Cluster Nb: 0–8 h: *p* = 0.006, 0–48 h: *p* = 0.007; Area: 0–8 h: *p* = 0.02, 0–48 h: *p* < 0.001; Intensity: 0–8 h: *p* = 0.04, 0–48 h: *p* < 0.001. ***E***, Example traces of α2 GABA_A_R trajectories at extrasynaptic (extra) and synaptic (syn) sites under control condition (t0) or after 8 or 48 h of 4-AP application. Scale bar, 0.25 µm. ***F***, Quantification of α2 GABA_A_R diffusion coefficients after 8 h of 4-AP exposure. Extra: t0 *n* = 1230 QDs, 4AP 8 h *n* = 1855 QDs, *p* = 3.4 × 10^−6^. Syn: t0 *n* = 281 QDs, 8 h *n* = 378 QDs, *p* = 0.2; 3 cultures. ***G***, Quantification of explored area EA of α2 GABA_A_R after 8 h of 4-AP application. Extra: t0 *n* = 3402 QDs, 8 h *n* = 2454 QDs, *p* = 3.2 × 10^−23^. Syn: t0 *n* = 843 QDs, 8 h *n* = 984 QDs, *p* = 0.02. ***H***, Quantification of α2 GABA_A_R diffusion coefficients after 48 h of 4-AP exposure. Extra: t0 *n* = 687 QDs, 4AP 48 h *n* = 1611 QDs, *p* = 0.4. Syn: t0 *n* = 73 QDs, 48 h *n* = 46 QDs, *p* = 1.6 × 10^−4^. ***I***, Quantification of explored area EA of α2 GABA_A_R after 48 h of 4-AP application. Extra: t0 *n* = 2061 QDs, 48 h *n* = 546 QDs, *p* = 2.9 × 10^−6^. Syn; t0 *n* = 219 QDs, 48 h *n* = 74 QDs, *p* = 6.6 × 10^−7^. ***J***, Quantification of α2 GABA_A_R dwell time DT after 8 or 48 h of 4-AP application. Calculations were done for trapped or passing QDs at inhibitory synapses. Trapped: t0: *n* = 130 QDs, 8 h: *n* = 194 QDs, *p* = 0.007; t0: *n* = 85 QDs, 48 h: *n* = 51 QDs, *p* = 0.02; Passing: t0: *n* = 91 QDs, 8 h: *n* = 161 QDs, *p* < 0.001; t0: *n* = 91 QDs, 48 h: *n* = 31 QDs, *p* = 0.6. In ***B–D*** and ***J***, data are presented as mean ± SEM. *, *p* ≤ 0.05; **, *p* ≤ 0.01; ***, *p* ≤ 0.001 (Mann–Whitney rank sum test). In ***F–I***, data are presented as median values ± 25%–75% IQR. *, *p* ≤ 0.05; ***, *p* ≤ 0.001 (Kolmogorov–Smirnov test). In all graphs, values were normalized to the corresponding control values.

It has been reported that gephyrin clustering is also influenced by the ERK1/2 pathway. We thus assessed whether ERK1/2 signaling influences gephyrin cluster size during chronic changes in network activity. Transgenic expression of eGFP-S268E gephyrin mutant renders gephyrin scaffold insensitive to the ERK1/2 signaling pathway (Tyagarajan et al., 2013). We therefore transfected cultured neurons with eGFP-S268E mutant and treated them with 4-AP for 8 or 48 h. Immunocytochemical analysis showed an increase in eGFP-S268E mutant cluster size after 4-AP treatment (Fig. 6*A*). Quantification of changes in eGFP-S268E cluster intensity confirmed an increase of 1.6- and 2.2-fold after 8 and 48 h of 4-AP treatment, respectively (Fig. 6*B*). This was associated with increases of 1.2- and 1.3-fold in eGFP-S268E cluster size after 8 and 48 h of 4-AP treatment (Fig. 6*B*). Analysis for α2 GABA_A_R cluster intensity at synapses and at extrasynaptic sites showed a respective 2.2- and 1.8-fold increase after 48 h of 4-AP treatment, but not after 8 h (Fig. 6*C*,*D*). We conclude that the eGFP-S268E mutant is not required for the activity-dependent recruitment of gephyrin and GABA_A_R within synaptic and extrasynaptic clusters. We wondered whether 4-AP induced chronic activity would impact the surface diffusion of GABA_A_Rs. We checked α2 GABA_A_R diffusion coefficients after 8 or 48 h of 4-AP. Individual receptor trajectories for extrasynaptic and synaptic α2 GABA_A_R suggested increased confinement after 48 h of enhanced activity (Fig. 6*E*). The α2 diffusion coefficients and explored area were increased by 1.2- and 1.4-fold for extrasynaptic receptors after 8 h of 4-AP (Fig. 6*F*,*G*). However, 48 h after 4-AP application, extrasynaptic receptors diffusion coefficients were unchanged (Fig 6*H*), whereas QDs were more confined at extrasynaptic sites (Fig. 6*I*). Interestingly, 48 h of 4-AP treatment reduced synaptic α2 GABA_A_R diffusion coefficients and explored areas at eGFP-S268E synapses (Fig. 6*H*,*I*), as observed at synapses containing eGFP-WT (Fig. 4*I*,*J*). In agreement with an increased number of α2 GABA_A_Rs at synapses, α2 dwell time increased at eGFP-S268E synapses (Fig. 6*J*).

Therefore, we conclude that although ERK1/2 signaling is not necessary for the activity-dependent regulation of the diffusive behavior of synaptic GABA_A_Rs, it controls the mobility of receptors at extrasynaptic sites. These observations further confirm that synaptic and extrasynaptic receptor pools are independently regulated, and that adaptations observed at GABAergic postsynapses are independent of the ERK1/2 pathway.

### GSK3β phosphorylation of gephyrin facilitates GABA_A_R diffusion after activity change

**Figure 7.**
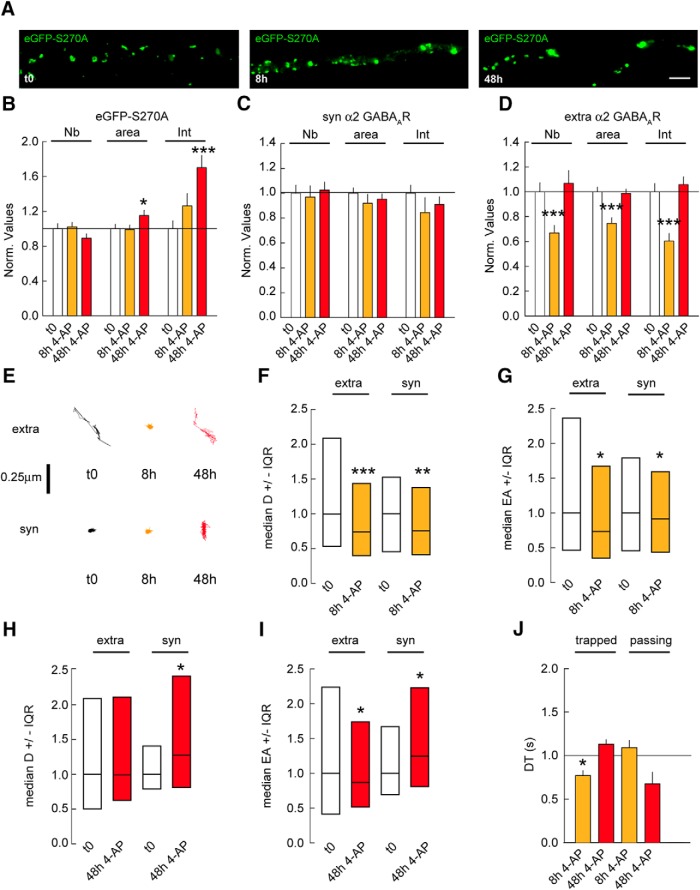
GSK3β pathway influences gephyrin scaffold and GABA_A_Rs after changes in chronic activity. ***A***, Morphology of neuron transfected with eGFP-S270A under control condition (t0) or in the presence of 4-AP after 8 or 48 h. Scale bar, 10 µm. ***B***, Quantification of eGFP-S270A clusters after 8 or 48 h of 4-AP application. t0 *n* = 43 cells, 8 h *n* = 50 cells; 48 h *n* = 50 cells, 3 cultures. Cluster Nb: 0–8 h: *p* = 0.8, 0–48 h: *p* = 0.14; Area: 0–8 h: Mann–Whitney test *p* = 0.7, 0–48 h: *p* = 0.04; Intensity: 0–8 h: *p* = 0.12, 0–48 h: *p* < 0.001. ***C***, Quantification of synaptic α2 GABA_A_R clusters after 8 and 48 h of 4-AP compared with mock treated control. t0 *n* = 40 cells, 8 h *n* = 47 cells; t0: *n* = 59 cells, 48 h *n* = 52 cells, 3–5 cultures. Cluster Nb: 0–8 h: *p* = 0.8, 0–48 h: *p* = 0.7; Area: 0–8 h: *p* = 0.14, 0–48 h: *p* = 0.6; Intensity: 0–8 h: *p* = 0.03, 0–48 h: *p* = 0.4. ***D***, Quantification of extrasynaptic α2 GABA_A_R clusters after 8 and 48 h of 4-AP compared with mock treated control. Cluster Nb: 0–8 h: *p* < 0.001, 0–48 h: *p* = 0.7; Area: 0–8 h: *p* < 0.001, 0–48 h: *p* = 0.7; Intensity: 0–8 h: *p* < 0.001, 0–48 h: *p* = 0.3. ***E***, Example traces of α2 GABA_A_R trajectories at extrasynaptic (extra) and synaptic (syn) sites under control conditions (t0) or after 8 or 48 h of 4-AP application. Scale bar, 0.25 µm. ***F***, Quantification of α2 GABA_A_R diffusion coefficients after 8 h of 4-AP exposure. Extra: t0 *n* = 1580 QDs, 4AP 8 h *n* = 1892 QDs, *p* = 1.4 × 10^−13^. Syn: t0 *n* = 229 QDs, 8 h *n* = 307 QDs, *p* = 8.8 × 10^−3^; 3 cultures. ***G***, Quantification of explored area EA of α2 GABA_A_R after 8 h of 4-AP application. Extra: t0 *n* = 4575 QDs, 8 h *n* = 4041 QDs, *p* = 0.02. Syn: t0 *n* = 687 QDs, 8 h *n* = 663 QDs, *p* = 0.04. ***H***, Quantification of α2 GABA_A_R diffusion coefficients after 48 h of 4-AP exposure. Extra: t0 *n* = 314 QDs, 4AP 48 h *n* = 338 QDs, *p* = 0.05. Syn: t0 *n* = 46 QDs, 48 h *n* = 51 QDs, *p* = 0.04. 3 cultures. ***I***, Quantification of explored area EA of α2 GABA_A_R after 48 h of 4-AP application. Extra: t0 *n* = 939 QDs, 48 h *n* = 771 QDs, *p* = 0.02. Syn; t0 *n* = 138 QDs, 48 h *n* = 153 QDs, *p* = 0.04. ***J***, Quantification of α2 GABA_A_R dwell time DT after 8 or 48 h of 4-AP application. Calculations were done for trapped or passing QDs at inhibitory synapses. Trapped: t0: *n* = 82 QDs, 8 h: *n* = 97 QDs, *p* = 0.04; t0: *n* = 191 QDs, 48 h: *n* = 45 QDs, *p* = 0.5; Passing: t0: *n* = 104 QDs, 8 h: *n* = 131 QDs, *p* = 0.5; t0: *n* = 211 QDs, 48 h: *n* = 23 QDs, *p* = 0.1. In ***B–D*** and ***J***, data are presented as mean ± SEM. *, *p* ≤ 0.05; ***, *p* ≤ 0.001 (Mann–Whitney rank sum test). In ***F–I***, data are presented as median values ± 25%–75% IQR. *, *p* ≤ 0.05; **, *p* ≤ 0.01; ***, *p* ≤ 0.001 (Kolmogorov–Smirnov test). In all graphs, values were normalized to the corresponding control values.

It has been reported that the GSK3β signaling pathway postsynaptically regulates the density and size of GABAergic synapses via gephyrin phosphorylation. Pharmacological blockade of the GSK3β pathway or expression of the S270A gephyrin mutant is sufficient to increase gephyrin cluster size (Tyagarajan et al., 2011). Hence, it is plausible that the GSK3β pathway acts in addition to PKA and CaMKIIα signaling to regulate homeostatic adaptations at GABAergic synapses. To address this question, we treated neurons transfected with eGFP-S270A gephyrin mutant with 4-AP for 8 and 48 h. Morphologic characterization showed that the GSK3β signaling is not essential for gephyrin accumulation at synapses on chronic changes in activity (Fig. 7*A*,*B*). In contrast, the eGFP-S270A mutant fully abolished the synaptic and extrasynaptic increase in α2 GABA_A_R clustering after 48 h of 4-AP application (Fig. 7*C*,*D*). After 8 h of 4-AP treatment, extrasynaptic α2 GABA_A_R cluster density, size, and intensity were respectively reduced by 1.4-, 1.2-, and 1.4-fold in eGFP-S270A–expressing cells (Fig. 7*D*). These results implicate the GSK3β pathway in the regulation of activity-induced GABA_A_R clustering at both synaptic and extrasynaptic sites.

If the GSK3β signaling is important for GABA_A_Rs accumulation at synapses in response to chronic changes in activity, then eGFP-S270A mutant expression should have no impact on α2 diffusion rates. However, 8 h after 4-AP application, α2 diffusion coefficients and explored areas were reduced by 1.3- and 1.1-fold at synaptic sites (Fig. 7*E–G*). This increased confinement was counterbalanced by a decrease in the time trapped receptors spent at synapses (Fig. 7*J*), explaining why α2 clustering was unchanged at synapses after 8 h of 4-AP application. On the other hand, 48 h of 4-AP application increased α2 diffusion coefficients by 1.3-fold as well as explored areas at synaptic sites (Fig. 7*E*,*H*,*I*). This was, however, not accompanied by a change in synaptic receptor dwell time (Fig. 7*J*). The reduction of extrasynaptic α2 clustering coincided with a 1.3-fold reduced explored area in eGFP-S270A–expressing cells after 8 h of 4-AP (Fig. 7*E–G*). Nevertheless, 48 h after 4-AP application, α2 diffusion coefficients and explored areas returned to baseline levels at extrasynaptic sites (Fig. 7*H*,*I*). These observations are consistent with the receptor clustering returning to control levels at extrasynaptic sites after 48 h of 4-AP (Fig. 7*D*). Altogether, these results show that GSK3β signaling in addition to PKA and CaMKIIα pathways tune GABA_A_Rs at synapses in response to chronic changes in activity.

### Impairment of PKA, CAMKIIα, and GSK3β phosphorylation of gephyrin abolishes the activity-dependent regulation of GABA_A_Rs mobility

The analysis of the SSA and S270A mutants indicated that PKA, CAMKIIα, and GSK3β phosphorylation of gephyrin have complementary effects on gephyrin and α2 GABA_A_Rs clustering in conditions of synaptic plasticity. To show it more directly, we generated eGFP-SSA/S270A mutant, expressed it in hippocampal neurons, and treated the neurons for 8 or 48 h with 4-AP.

**Figure 8.**
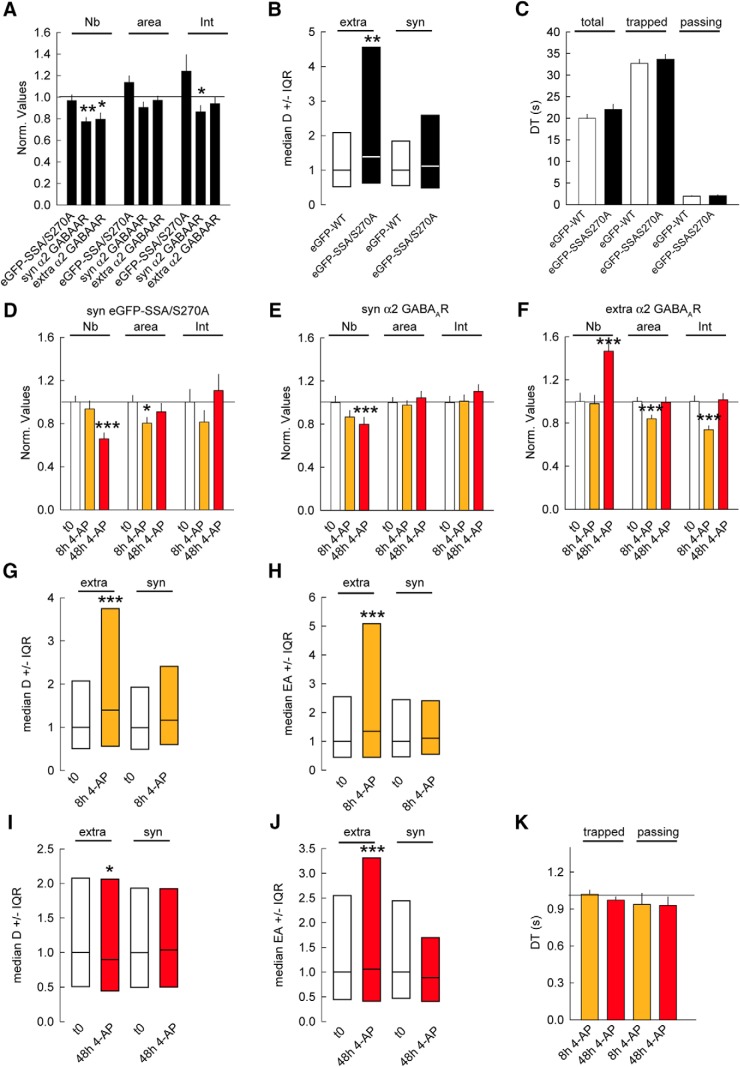
PKA, CAMKIIα, and GSK3β pathways are required to tune the inhibitory synapse. ***A***, Quantifications of synaptic eGFP-SSA/S270 clusters and synaptic (α2 syn) and extrasynaptic (α2 extra) α2 GABA_A_R clusters in relation to eGFP-WT show minor impact of the mutant under control condition. eGFP-WT *n* = 58 cells, eGFP-SSA/S270A *n* = 62 cells, 3 cultures. eGFP-SSA: Cluster Nb: *p* = 0.6; Area: *p* = 0.1; Intensity: *p* = 0.7. α2 syn: Cluster Nb: *p* = 0.001; Area: *p* = 0.1; Intensity: *p* = 0.02. α2 extra: Cluster Nb: *p* = 0.03; Area: *p* = 0.5; Intensity: *p* = 0.2. ***B***, Quantification of median diffusion coefficient D of α2 GABA_A_R in neurons expressing eGFP-WT or eGFP-SSA/S270A under control condition. Extra: WT *n* = 823 QDs, SSA/S270A *n* = 786 QDs, *p* = 0.004; Syn: WT *n* = 261 QDs, SSA/S270A *n* = 211 QDs, *p* = 0.3, 2 cultures. ***C***, Quantification of α2 GABA_A_R dwell time DT at synaptic sites in neurons expressing either eGFP-WT or eGFP-SSA/S270A. Calculations were done for all QDs (total), (trapped), or (passing) QDs at inhibitory synapses. No significant differences were found between eGFP-WT and eGFP-SSA/S270A. Total: WT *n* = 165 QDs, SSA/S270A *n* = 183 QDs, *p* = 0.1; Trapped: WT *n* = 95 QDs, SSA/S270A *n* = 116 QDs, *p* = 0.5; Passing: WT *n* = 70 QDs, SSA/S270A *n* = 67 QDs, *p* = 0.2. ***D***, Quantification of eGFP-SSA/S270A clusters after 8 or 48 h of 4-AP application. t0 *n* = 53 cells, 8 h *n* = 45 cells, 48 h *n* = 51 cells, 3 cultures. Cluster Nb: 0–8 h: *p* = 0.3, 0–48 h: *p* < 0.001; Area: 0–8 h: *p* = 0.03, 0–48 h: *p* = 0.2; Intensity: 0–8 h: *p* = 0.3, 0–48 h: *p* = 0.9. ***E***, Quantification of synaptic α2 GABA_A_R clusters after 8 and 48 h of 4-AP compared with mock treated control. t0 *n* = 49 cells, 8 h *n* = 49 cells, 48 h *n* = 39 cells, 3 cultures. Cluster Nb: 0–8 h: *p* = 0.2, 0–48 h: *p* < 0.001; Area: 0–8 h: *p* = 0.8, 0–48 h: *p* = 0.6; Intensity: 0–8 h: *p* = 0.2, 0–48 h: *p* = 0.9. ***F***, Quantification of extrasynaptic α2 GABA_A_R clusters after 8 and 48 h of 4-AP compared with mock treated control. Cluster Nb: 0–8 h: *p* = 0.8, 0–48 h: *p* = 0.001; Area: 0–8 h: *p* < 0.001, 0–48 h: *p* = 0.7; Intensity: 0–8 h: *p* = 0.8, 0–48 h: *p* = 0.8. ***G***, Quantification of α2 GABA_A_R diffusion coefficients after 8 h of 4-AP exposure. Extra: t0 *n* = 624 QDs, 4AP 8 h *n* = 421 QDs, *p* = 5.4 × 10^−7^. Syn: t0 *n* = 252 QDs, 8 h *n* = 173 QDs, *p* = 0.2, 2 cultures. ***H***, Quantification of explored area EA of α2 GABA_A_R after 8 h of 4-AP application. Extra: t0 *n* = 1869 QDs, 8 h *n* = 1092 QDs, *p* = 7.8 × 10^−14^. Syn: t0 *n* = 753 QDs, 8 h *n* = 516 QDs, *p* = 0.07. ***I***, Quantification of α2 GABA_A_R diffusion coefficients after 48 h of 4-AP exposure. Extra: t0 *n* = 624 QDs, 4AP 48 h *n* = 631 QDs, *p* = 0.04. Syn: t0 *n* = 252 QDs, 48 h *n* = 251 QDs, *p* = 0.8. 2 cultures. ***J***, Quantification of explored area EA of α2 GABA_A_R after 48 h of 4-AP application. Extra: t0 *n* = 1092 QDs, 48 h *n* = 1890 QDs, *p* = 1.5 × 10^−6^. Syn; t0 *n* = 558 QDs, 48 h *n* = 750 QDs, *p* = 0.3. ***K***, Quantification of α2 GABA_A_R dwell time DT after 8 or 48 h of 4-AP application. Calculations were done for trapped or passing QDs at inhibitory synapses. Trapped: t0: *n* = 116 QDs, 8 h: *n* = 84 QDs, 48 h: *n* = 43 QDs, 0–8 h: *p* = 0.2; 0–48 h: *p* = 0.02; Passing: t0: *n* = 67 QDs, 8 h: *n* = 46 QDs, 48 h: *n* = 43 QDs, 0–8 h: *p* = 0.2; 0–48 h: *p* = 0.1. In ***A***, ***C–F***, and ***K***, data are presented as mean ± SEM. *, *p* ≤ 0.05; ***, *p* ≤ 0.001 (Mann–Whitney rank sum test). In ***G–J***, data are presented as median values ± 25%–75% IQR. *, *p* ≤ 0.05; ***, *p* ≤ 0.001 (Kolmogorov–Smirnov test). In all graphs except ***C***, values were normalized to the corresponding control values.

We found that overexpressing eGFP-SSA/S270A increased eGFP cluster size and intensity (Fig. 8*A*). The gephyrin cluster growth was, however, not accompanied by synaptic recruitment of α2 GABA_A_Rs (Fig. 8*A*). Although the density of α2 GABA_A_Rs clusters was reduced in eGFP-SSA/S270A–transfected cells, there was no major impact of the mutant on α2 GABA_A_Rs cluster size and intensity at synaptic and extrasynaptic sites (Fig. 8*A*).

We then characterized α2 GABA_A_R diffusion in SSA/S270A-transfected neurons. Diffusion coefficients showed a 1.4-fold increase for extrasynaptic receptors and no significant change for synaptic receptors (Fig. 8*B*). This effect was consistent with the observation that α2 GABA_A_R spent the same time at eGFP-SSA/S270A and eGFP-WT synapses (Fig. 8*C*). Therefore the eGFP-SSA/S270A mutant can recapitulate many of the observed phenotypes seen with SSA or S270A individual mutations.

We then characterized how chronic activity affects eGFP-SSA/S270A mutant behavior. Although the extrasynaptic GABA_A_Rs cluster density increased after 48 h of 4-AP in eGFP-SSA/S270A transfected cells, the triple mutant prevented the synaptic increase in gephyrin and GABA_A_Rs cluster size and intensity in response to 4-AP treatment (Fig. 8*D–F*). The diffusion coefficient and explored area of α2 GABA_A_Rs showed no change after 8 or 48 h of 4-AP application (Fig. 8*G–J*). There was also no impact on receptor dwell time at synapses after chronic changes in activity (Fig. 8*K*).

Our results uncover a role for several signaling pathways in chronic activity-dependent modulation of gephyrin clustering and GABA_A_Rs surface diffusion at synapses. Our data also show that distinct signaling pathways regulate synaptic and extrasynaptic receptor clustering. Together, these results identify a novel role of GSK3β signaling in the regulation of extrasynaptic receptor surface trafficking and GSK3β, PKA, and CaMKIIα pathways in facilitating adaptations of synaptic receptors.

## Discussion

In the current study, we investigate the molecular basis for gephyrin scaffold–induced GABA_A_R membrane dynamics. We identify a novel role for gephyrin posttranslational modification involving phosphorylation and dephosphorylation in regulating GABA_A_R lateral diffusion. By tracking α2 GABA_A_Rs within and outside synaptic sites using QD-SPT, we demonstrate that gephyrin phosphorylation by ERK1/2 at S268, and inhibition of GSK3β phosphorylation on gephyrin at S270, while exhibiting opposite effects on synaptic morphology, similarly influence GABA_A_R diffusion properties. We analyze gephyrin scaffold organization at the nanoscale level using PALM and uncover that phosphorylation also controls gephyrin molecule packing.

Over the past decade, several independent studies have documented changes in lateral diffusion of GABA_A_Rs after pharmacological alteration of neuronal function within a time scale of minutes to a few hours (Lévi et al., 2008; Bannai et al., 2009; Niwa et al., 2012; Petrini et al., 2014). 4-AP application within minutes induces NMDA receptor–mediated calcium influx and calcineurin activation, leading to dephosphorylation of the GABA_A_R γ2 subunit S327 residue (Wang et al., 2003). In this context, an increase in GABA_A_R diffusion constraint results from receptor dephosphorylation, whereas gephyrin scaffold loss is a secondary effect in response to receptor dispersal (Niwa et al., 2012). We identify gephyrin phosphorylation as an essential facilitator of GABA_A_R diffusion dynamics in response to chronic changes in activity. More specifically, we identify a central role for PKA and CaMKIIα pathways along with GSK3β signaling in phosphorylating gephyrin to regulate activity-dependent inhibitory synapse remodeling.

### Structure of the gephyrin scaffold requires phosphoregulation of gephyrin molecules

At GABAergic synapses, the role of phosphorylation for gephyrin scaffold compaction has yet to be reported. The fluorescence microscopy data (Fig. 1*B*) inform us about average area and intensity per cluster. PALM microscopy informs us about the actual density of molecules per surface area (Fig. 3). The number of molecules per synapse using PALM imaging can be roughly estimated by multiplying the mean surface area of the cluster by the density of gephyrin molecules per surface unit. Values of ∼212, 156, and 220 were found for the gephyrin WT, S268E, and S270A respectively. Interestingly, these estimations are consistent with the measurements of the mean cluster fluorescence intensity for the S268E and S270A mutants.

The hexameric gephyrin lattice model was proposed based on G and E domain crystal structures available at the time. However, in recent years, atomic force microscopy (AFM) and small-angle X-ray scattering (SAXS) structure of full-length gephyrin has shown that gephyrin only exists as trimers, as individual E domains are in an open extended confirmation (Sander et al., 2013). Pennacchietti et al. (2017) have shown that after iLTP, gephyrin reorganizes itself into distinct subsynaptic nanodomains. Full-length gephyrin can exist in open or closed confirmations based on the linker domain folding (Sander et al., 2013). All the gephyrin phosphorylation sites have been mapped to the linker domain, suggesting that phosphorylation is a strong candidate for determining open and closed states within gephyrin nanodomains. This could in turn determine the distance between two nanodomains and/or total number of nanodomains within a given synapse.

### Gephyrin-independent GABA_A_R adaptations at synaptic sites

It has long been assumed that alterations in GABA_A_R and/or gephyrin cluster intensity are indicative of the number of molecules found at the synapse, and thereby a direct correlate for changes in synapse structure and function. Here we report that disrupting gephyrin scaffold via the expression of the eGFP-DN mutant does not increase the diffusion properties of GABA_A_Rs at synaptic sites. This observation was unexpected, as loss of the scaffolding apparatus should have increased receptor diffusion also at synaptic sites. It has been reported that eGFP-DN expression significantly reduces mIPSC amplitude and frequency, without leading to a complete loss of GABAergic synaptic transmission (Ghosh et al., 2016). Our observation suggests that a pool of gephyrin-independent GABA_A_Rs are present in neurons. Recently, the GIT1/βPIX/Rac1/PAK signaling pathway was shown to contribute to GABAergic transmission. βPIX is a guanine nucleotide exchange factor (GEF) for Rac1 activating PAK and contributes to GABA_A_R stability (Smith et al., 2014). Similar signaling mechanisms could be operational even in the absence of gephyrin scaffold to maintain the membrane pool of GABA_A_Rs.

### Independent behavior of GABA_A_Rs at synaptic and extrasynaptic sites

Postsynaptic receptor trapping is adaptable depending on phosphorylation events that impinge on scaffold–scaffold or receptor–scaffold interactions (Choquet and Triller, 2013). It became clear with the development of SPT approaches that receptors are also hindered in their diffusion outside synapses via molecular crowding but also through specific protein–protein interactions. A receptor–gephyrin interaction outside inhibitory synapses has been reported earlier (Ehrensperger et al., 2007). GABA_A_Rs also colocalize and interact with clathrin-enriched endocytic zones (EZs) that are mostly localized extrasynaptically (Smith et al., 2012). Receptors in EZs do not necessarily undergo internalization. They can be part of a reserve pool of receptors rapidly available upon increase in synaptic activity (Petrini et al., 2014). Conversely, the GABA_A_R–AP2 interaction within EZs has been shown to indirectly control receptor mobility and number at synapses (Smith et al., 2012).

However, our data show independent behavior of GABA_A_Rs at synaptic and extrasynaptic sites. After 8 h of 4-AP treatment, α2 GABA_A_Rs were confined at extrasynaptic sites without influencing the diffusion property of synaptic receptors. In contrast, after 48 h of 4-AP treatment, α2 GABA_A_R confinement at extrasynaptic sites was ceased, and this was followed by an increase in receptor confinement at synapses, suggesting that GABA_A_R retention at extrasynaptic sites prevents their synaptic capture/accumulation. However, after 8 h of 4-AP treatment, neurons expressing eGFP-S268E mutant show a reduction in receptor confinement at extrasynaptic locations, without affecting synaptic receptor diffusion. Therefore, removing diffusion constraints onto extrasynaptic GABA_A_Rs does not facilitate receptor recruitment at synapses. In addition, after 8 h of 4-AP treatment, neurons expressing eGFP-S270A mutant show increased confinement of GABA_A_Rs at both extrasynaptic and synaptic locations, indicating that confining GABA_A_Rs at extrasynaptic locations does not prevent diffusion-capture of receptors.

### Synaptic adaptation is facilitated by gephyrin phosphorylation

We present evidence for a biphasic model for activity-dependent plasticity at GABAergic postsynapse. Acute 4-AP treatment increases and chronic 4-AP treatment decreases α2 GABA_A_R lateral diffusion. The observed increase in GABA_A_R diffusion after acute 4-AP treatment can be explained by an increase in synaptic escape of receptors, leading to reduced postsynaptic clustering and dispersal of gephyrin molecules away from the synapse (Bannai et al., 2009). On the contrary, we show here that chronic 4-AP treatment leads to synaptic immobilization and recruitment of GABA_A_R α2 and gephyrin. These discrepancies are probably due to the distinct signaling pathways activated by the acute and chronic changes in activity. Short-term 4-AP application induces NMDAR-mediated calcium influx and calcineurin activation, leading to dephosphorylation of GABA_A_R γ2 subunit S327 residue (Bannai et al., 2009). In this context, the relief in GABA_A_R diffusion constraints arises from receptor dephosphorylation, whereas gephyrin loss is a consequence of receptor dispersal (Niwa et al., 2012). In contrast, we show that chronic changes in activity affect first the recruitment of gephyrin at synapses, and then allow the recruitment of GABA_A_Rs. PKA and CaMKIIα signaling act downstream of NMDA receptor to facilitate compensatory postsynaptic adaptations at GABAergic synapses (Flores et al., 2015). Our data extends this understanding by demonstrating a role for the GSK3β pathway in addition to PKA and CaMKIIα pathways in facilitating gephyrin scaffold organization of individual GABA_A_Rs after prolonged changes in activity.

## Synthesis

Reviewing Editor: Marlene Bartos, University of Freiburg Decisions are customarily a result of the Reviewing Editor and the peer reviewers coming together and discussing their recommendations until a consensus is reached. When revisions are invited, a fact-based synthesis statement explaining their decision and outlining what is needed to prepare a revision will be listed below. The following reviewer(s) agreed to reveal their identity: Laurent Groc, Andrea Barberis.

Reviewer #1:

In this study the authors have investigated whether phosphorylation of the main scaffolding protein gephyrin could regulates both the inhibitory scaffold organization and the surface dynamics of the GABAA receptors. To this aim, the authors combine immunocytochemistry, super-resolution PALM imaging and single particle tracking experiments on rat hippocampal cultured neurons transfected with mutants of gephyrin. The authors have transfected the ERK1/2 phosphomimetic S268E and the GSK3β-dependent inactive mutant S270A to investigate the cluster's properties of the α2 GABAA receptors. These mutants are known to modulate the gephyrin scaffold. E270A increases while S268E decreases the number and the size of the gephyrin clusters. This study is of great interest, shedding new lights on the regulation of GABAa receptor trafficking. Several points should be addressed in a revised manuscript.

Major comments:

In figure 1, the authors have only shown the quantification of the cluster intensity. As one can clearly see differences in the staining of the α2 GABAA receptors, a more detailed analysis is needed (i.e. size and number of clusters). This analysis has also to be done to characterize the colocalization between VGAT and the mutants of gephyrin. This general comment applies for all the immunocytochemistry experiments.

In figure 2, I am very surprised regarding the quantum dots experiment and the GABAA receptors surface dynamics. How can the median D and EA be almost identical in control conditions? Don't the authors expect significant differences between extrasynaptic vs synaptic areas ? In eGFP-DN transfected neurons, the authors used the presynaptic markers VGAT -oyster550 to identify synaptic sites but as the gephyrin has a diffuse expression is it still relevant to discriminate between extrasynaptic and synaptic trajectories? Please, clarify these points.

My major concern is the quantum dots experiments performed on the neurons transfected with the E270A and S268E gephyrin mutants. In both experiments the median D and EA is increased at extrasynaptic and synaptic sites. Therefore, there is no correlation between the surface dynamics of the α2 GABAA receptors and the properties of the gephyrin scaffold. Please, clarify these points.

The super-resolution PALM microscopy experiments have confirmed that the S268E and S270A mutants differentially regulate the inhibitory synaptic areas. The increased synaptic areas are correlated with a decreased molecular density in neurons transfected with S270A mutants. On the other hand, in S268E transfected neurons, the decreased synaptic areas are correlated with an increased molecular density. This is in total contraction with the results illustrated in figure 1B where the authors showed an increase (mutant S270A) and a decrease (mutant S268E) in the cluster intensity. The authors should, at least, comment this point.

Regarding the 4-AP treatment experiments, the authors claim that they observed a decrease in GABAA receptors at 48h time point and not before. My main concern is that the only experiment performed before has been done at 8h. How can they rule out an earlier effect of the 4-AP without a more detailed timescale (i.e. 24h, 36h)?

What is the effect of the 4-AP treatment at 8h on the gephyrin and GABAA receptors clusters in neurons transfected with the SSA mutant?

Why are the QD experiments illustrated as a whole for the SSA mutant (with or without 4-AP) and not as extrasynaptic vs. synaptic trajectories?

The authors claim that the 4-AP treatment can override the S268E mutant phenotype. This is partly true as the GABAA receptors diffusion properties are still different between the mutant and the WT. The authors only illustrated the median diffusion coefficient. What about the effect of the 4-AP treatment on the median explored area and the dwell time?

To fully support their conclusions, the authors could perform additional experiments mixing gephyrin mutants and pharmacological tools (i.e. blockade of GSK3β pathways, ERK inhibitors) together with the 4-AP treatment. Otherwise, the authors could tune down some of their conclusions, and further discuss these aspects.

Minor comments:

-The authors should check the scale in figures 6D/7D and 4D

-The authors should increase the number of experiments in figure 7F. It is striking that there is no significant effect of the 4-AP on the diffusion coefficient values.

Reviewer #2:

The manuscript advances our knowledge on how the phosphorylation state of gephyrin modulates the GABAA receptor-gephyrin interaction. In particular this is studied in long term plasticity paradigms (48 h). This can contribute clarifying post-synaptic mechanisms of plasticity, a relevant but poorly studied topic.

Major points:

1) Several sentences are scientifically imprecise or inaccurate through the whole manuscript. Some examples are listed below.

First sentence of the abstract: “Synaptic plasticity relies on the rapid change in neurotransmitter receptor number in the postsynaptic membrane, which is regulated ‐ apart from endocytosis and exocytosis - by lateral diffusion”. While I may understand the message the Authors wants to convey it has to be remarked that synaptic plasticity does not only rely of “rapid change of neurotransmitter receptor number”: While there are example of short term plasticity in which receptor are exchanged in tens of milliseconds, in many forms of plasticity, receptors are rearranged in the time range of minutes Bannai et al., 2009; Petrini et al., 2014). Also, the definition “postsynaptic membrane” should be substituted by “postsynaptic sites” or “postsynaptic areas”. Typically it is assumed that “postsynaptic membrane” also includes perisynaptic and extrasynaptic sites.

Introduction, first two lines: “...essential role in information transfer between neuronal networks”: GABAA-mediated information transfer conventionally occurs between two neurons not between networks. If the author really meant that information can be transferred “between neuronal networks” please specify how, and why it is relevant in this context.

Page 3 line 9 from bottom: “...phosphorylation of PKA...”: as far as I know, phosphorylation of gephyrin (S303) by PKA has not been implicated in the plasticity described in Flores et al., 2015. In that study it is stated that “phosphorylation of gephyrin on S305 site (by CaMKII) is both sufficient and necessary to promote inhibitory synapse formation in response to neuronal activity”. Please clarify.

Page 11, line 11 from bottom: “GABAARs undergo rapid Brownian-type motion at extrasynaptic sites, while...”. This sentence is misleading since it passes the message GABAAR receptors diffuse according the Brownian motion rules only at extrasynaptic sites. Maybe the Authors meant that at extrasynaptic space GABAAR obey to the rules of “free Brownian diffusion” while at synapses GABAAR to those of “confined diffusion”.

Page 20, line 7 from bottom: Bannai et al., 2009 did not demonstrate that S327 is involved in calcineurin-dependent dephosphorylation. The paper the Authors are referring to is actually “Wang et al., 2003”, and is only cited in the discussion of Bannai et al., 2009. Please pay attention to the appropriateness of the citations quoted.

Page 21, line 6 from bottom: “After 8 h of 4-AP treatment α2 GABAAR were confined to extrasynaptic locations without influencing the diffusion property of synaptic receptors”. It is obscure how receptors can be confined at extrasynaptic sites since receptors in the extrasynaptic space undergo free diffusion. Did the Author mean that alpha2-containing receptors are actively excluded from the synaptic areas? How can receptors be retained at extrasynaptic sites? Which the forces involved in this “retention” are? Please clarify.

2) The term “homeostatic” in some cases is used incorrectly. For instance, in the introduction, the Authors state that homeostatic adaptations take place in Bannai et al., 2009. In contrast that study shows a short term reduction of inhibition following a stimulation protocol (with 4-AP), that has nothing to do with homeostasis. Indeed, it is raised the possibility that transient reduction of inhibition could favor LTP onset, meaning that there is no parallel change between excitability and inhibition (i.e. the definition of homeostatic plasticity). Even less “homeostatic” is the paradigm reported in Petrini et al., 2014 in which, in contrast, they report an anti-homeostatic process where potentiation of inhibition is accompanied by reduction of excitation. Similar concept has been suggested in Marsden et al., 2010.

Bannai et al., 2009 and Petrini et a., 2014 are very good examples of how lateral diffusion tune the synaptic inhibitory strength, but not in a homeostatic way.

My point here is that “homeostatic change” is not a synonym of “synaptic plasticity” or “chronic change”. Homeostatic plasticity is a particular type of plasticity in which synaptic changes maintain the “excitability equilibrium” of neurons or neuronal networks.

3) In the introduction, the Authors state that in Saliba et al., 2012 there is enhanced neurotransmission. In contrast, in that study, there is no enhanced neurotransmission but rather increased tonic conductance.

4) The terms “scaffold” or “scaffolding” are not synonyms of “synaptic clustering of gephyrin”, as they are often used in this manuscript (e.g. page 3, line 7 from top; page 10 line 7 from bottom; page 18 first line).

5) The long term effect of 4-AP treatment on gephyrin clustering and GABAAR mobility is opposite with respect to that observed in acute by Bannai et al., 2009. While I think that this “acute” versus “chronic” effect is very interesting, I didn't fully understand the “molecular” explanation provided for this discrepancy. Please clarify.

6) The S268E and S270A gephyrin synaptic clusters are reported to show different density. The Authors hypothesize that the increased density of S268E clusters is responsible for the reduction in GABAAR mobility at synapses. Although in the Author's view this explanation is rather straightforward, this theory needs to be further discussed in the light of the works that addressed the molecular organization of gephyrin at synapse and/or the stoichiometry gephyrin-GABAAR (e.g. Bannai et al., 2009). Indeed, according to the current theory, gephyrin molecules trimerize through the G domain and, subsequently, trimers dimerize through the E domain to form examers. Thus an “exameric” monolayer latex matrix is formed beneath the synapse. Furthermore, receptors should interact with gephyrin in stoichiometric ratio 1:1. Taking into account these data, how the Authors think an increase of gephyrin could affect the availability of gephyrin molecules at synapses? Indeed according to what is known, gephyrin cannot be further packed (in 2D), unless the authors hypothesize the formation of a different distribution/molecular organization. Alternatively (or in addition) it could be hypothesized a change in the GABAAR-gephyrin stoichiometry. I'm not dogmatic on this, but possible scenarios should be provided (and discussed with respect to previous literature).

7) The authors should better detail the clustering algorithm used for identifying the gephyrin clusters in super-resolution experiments. Does the observed differences of cluster area/density are algorithm-clustering-dependent?

Minor points:

The text must be improved from the grammatical and syntactical point of view. In its present form, the manuscript is difficult to read and the message is obscured. There are also “here and there” some repetitions. I strongly recommend a substantial critical editing of the manuscript by a professional manuscript editor.

1) Page 10, line 7 from top: “simulating phosphorylation....”: maybe the authors meant “mimicking phosphorylation”

2) Page 11, lines 9 and 10: “similar reduction” and “similar increase”. Specify similar to what.

3) Page 12, line 9 from bottom: “Thus, we concluded the”. Maybe the Authors meant: “Thus we concluded that”. Same issue at page 13, line 10 from bottom

4) Page 12 line 5 from bottom: “To test this idea”. Maybe the Authors meant: “To test this hypothesis” Same issue at page 15, line 11

5) Page 13 line 4: “then we must also notice”, Although a understand what the author mean I would rephrase it as: “then we should also measure”

6) Page 13 last line: “PALM images can indicate”. Instead of “indicate” I would use “resolve”.

7) Page 15, line 10 from bottom: “increases by 1.95 and 2.3 fold after 8 h or 48 h 4-AP treatment”. Please make “and”/“or” coherent. At the end of the sentence “respectively” should be used.

8) Page 17, line 10: “Our data identifies”. “Our data identify”.

9) Page 19, line “: “It is entirely possible”. I would change it with “It is plausible”, for instance.

10) Page 20, line 10: “determines gephyrin packing”. “Controls” or “modulates”, seem to me more appropriate than “determines”.

## Author Response

Please find below our response to reviewers' comments.

Reviewer #1:

This study is of great interest, shedding new lights on the regulation of GABAa receptor trafficking.

We are thankful to the reviewer for his/her recognition of the accomplished work.

Major comments:

In figure 1, the authors have only shown the quantification of the cluster intensity. As one can clearly see differences in the staining of the α2 GABAA receptors, a more detailed analysis is needed (i.e. size and number of clusters). This analysis has also to be done to characterize the colocalization between VGAT and the mutants of gephyrin. This general comment applies for all the immunocytochemistry experiments.

We have now reanalyzed and presented the data as requested by the reviewer for all our ICC experiments.

In figure 2, I am very surprised regarding the quantum dots experiment and the GABAA receptors surface dynamics. How can the median D and EA be almost identical in control conditions? Don't the authors expect significant differences between extrasynaptic vs synaptic areas ? fig 1 e and 1b.

It is difficult to measure receptor diffusion at synapses using VGAT or gephyrin clusters as synaptic masks. VGAT punctae are larger than gephyrin clusters meaning that VGAT masks may extend to regions that are not associated with the active zone where receptor mobility could be faster. Second, we have reported in the past that only a small proportion of GABAARs crossing inhibitory synapses are actually captured by the scaffold at rest (Renner et al., 2012). This could explain why it is difficult to have a statistical difference between synaptic and extrasynaptic receptors.

In eGFP-DN transfected neurons, the authors used the presynaptic markers VGAT -oyster550 to identify synaptic sites but as the gephyrin has a diffuse expression is it still relevant to discriminate between extrasynaptic and synaptic trajectories? Please, clarify these points.

We believe it is necessary to separate extrasynaptic and synaptic trajectories in eGFP-DN neurons as or results show that diffuse gephyrin molecules can slow down receptors at synapses as well as at extrasynaptic sites. We have now added a sentence (pg 12 line 23) to clarify the text better: These observations support the notion that gephyrin not only slows down and confines GABAARs at synapses but also at extrasynaptic sites (Ehrensperger et al., 2007).

My major concern is the quantum dots experiments performed on the neurons transfected with the S270A and S268E gephyrin mutants. In both experiments the median D and EA is increased at extrasynaptic and synaptic sites. Therefore, there is no correlation between the surface dynamics of the α2 GABAA receptors and the properties of the gephyrin scaffold. Please, clarify these points.

Our data indicates that there is no correlation between the diffusion properties of the GABAAR in spite of the relative size difference between S268E and S270A gephyrin clusters. However, there is a strong correlation between gephyrin phosphorylation and cluster microdomain compaction. We show that S268E mutant decreased the size of gephyrin clusters by increasing the compaction of gephyrin molecules. Conversely, the S270A mutant increased the size of gephyrin clusters by increasing the spacing between gephyrin molecules. The compaction of the scaffold or the increased spacing between gephyrin molecules may perturb the trimeric organization of the gephyrin microdomains thereby altering gephyrin-receptor binding properties. However, we cannot exclude the possibility that the mutations impact directly receptor-binding properties independently of their effect on the mesh. We have now discussed this point in the manuscript (pg 15 line 13).

The super-resolution PALM microscopy experiments have confirmed that the S268E and S270A mutants differentially regulate the inhibitory synaptic areas. The increased synaptic areas are correlated with a decreased molecular density in neurons transfected with S270A mutants. On the other hand, in S268E transfected neurons, the decreased synaptic areas are correlated with an increased molecular density. This is in total contradiction with the results illustrated in figure 1B where the authors showed an increase (mutant S270A) and a decrease (mutant S268E) in the cluster intensity. The authors should, at least, comment this point.

The following paragraph has been added to the discussion section (pg 24 line 20): “The fluorescence microscopy data (Figure 1B) inform us about average area and intensity per cluster. PALM microscopy informs us about the actual density of molecules per surface area (Figure 3). The number of molecules per synapse using PALM imaging can be roughly estimated by multiplying the mean surface area of the cluster by the density of gephyrin molecules per surface unit. Values of ~ 212, 156 and 220 were found for the gephyrin WT, S268E and S270A respectively. Interestingly, these estimations are consistent with the measurements of the mean cluster fluorescence intensity for the S268E and S270A mutants.”

Regarding the 4-AP treatment experiments, the authors claim that they observed a decrease in GABAA receptors at 48h time point and not before. My main concern is that the only experiment performed before has been done at 8h. How can they rule out an earlier effect of the 4-AP without a more detailed timescale (i.e. 24h, 36h)?

We are sorry for the confusion. To our knowledge, our work is the first to analyze receptors lateral diffusion on a time scale of days. Combining SPT and ICC experiments is really time consuming. We consider that the outcome of analyzing additional time points is not critical for the message. We have therefore corrected the text throughout the manuscript to be more specific on the different time points being compared in order to prevent confusion.

What is the effect of the 4-AP treatment at 8h on the gephyrin and GABAA receptors clusters in neurons transfected with the SSA mutant?

We have performed additional experiments to address the reviewer question. Our results indicate that the SSA mutant blocks the increase in gephyrin clustering at synapses 8 h after 4-AP treatment. The effect is maintained 48h after 4-AP application. We further showed that the SSA mutant blocked the 4-AP induced recruitment of α2 GABAAR at synapses but not at extrasynaptic site.

Why are the QD experiments illustrated as a whole for the SSA mutant (with or without 4-AP) and not as extrasynaptic vs. synaptic trajectories?

We have now amended the figure to show quantifications on both synaptic and extrasynaptic trajectories (Figure 5I-M). The following paragraph was added to the Results section (pg 89 line 25): “Similar to wild-type gephyrin, SSA reduced diffusion coefficient and surface exploration of α2 GABAARs at extrasynaptic sites after 8 h of 4-AP (Fig. 5I-J). This effect was maintained also after 48 h treatment (Fig. 5K-L). In contrast to wild-type gephyrin, SSA mutant increased α2 GABAAR confinement, and decreased resident time of GABAARs at synapses after 48 h of 4-AP (Fig. 5K-M). The passing α2 GABAARs remained unchanged at synapses after 8 h or 48 h of 4-AP (Fig. 5M). Hence, our results indicate that gephyrin scaffold reorganization via PKA and CaMKII α dependent phosphorylation at S303 and S305 is essential for GABAAR diffusion at synapses but not at extrasynaptic sites in response to chronic changes in activity.”

The authors claim that the 4-AP treatment can override the S268E mutant phenotype. This is partly true as the GABAA receptors diffusion properties are still different between the mutant and the WT. The authors only illustrated the median diffusion coefficient. What about the effect of the 4-AP treatment on the median explored area and the dwell time?

The effect of the 4-AP treatment on the median explored area and the mean dwell time have been added as requested (Figure 6I-J). They reveal that the S268E mutant does not prevent the 4-AP induced recruitment of gephyrin and GABAAR at synapses, and influences the lateral diffusion of the α2 subunit at extrasynaptic sites.

To fully support their conclusions, the authors could perform additional experiments mixing gephyrin mutants and pharmacological tools (i.e. blockade of GSK3β pathways, ERK inhibitors) together with the 4-AP treatment. Otherwise, the authors could tune down some of their conclusions, and further discuss these aspects.

We have now included new experiments in the revised manuscript (Figure 8). Knowing that the signaling pathways studied here - PKA, CAMKII or GSK3β - have a wide variety of targets, the pharmacological approach involving the use of inhibitors has been ruled out. We preferred instead to use a genetic approach to directly test the hypothesis that PKA, CAMKII and GSK3β phosphylation of gephyrin have complementary actions on the homeostatic regulation of the inhibitory synapse. We generated the eGFP-SSA/S270A mutant, expressed it in hippocampal neurons and treated the neurons for 8 h or 48 h with 4-AP. We found that, in basal activity conditions, the eGFP-SSA/S270A mutant exhibits normal clustering with no effect on GABAAR dwell time at synapses.

We then characterized the behavior of the triple mutant to chronic activity change, we found that the eGFP-SSA/S270A mutant prevented gephyrin and GABAARs cluster growth at synapses and extrasynaptically. In line with these observations, the diffusion coefficient and explored area of synaptic α2 GABAARs were not modified after 8 h or 48 h of 4-AP in SSAS270A expressing cells.

Based on these observations, we conclude that the eGFP-SSA/S270A mutant can recapitulate many of the observed phenotypes seen with SSA or S270A individual mutations. We thank the reviewer for her/his suggestion since these experiments strengthened our hypothesis of the contribution of several signaling pathways in chronic activity-dependent modulation of gephyrin clustering and GABAARs surface diffusion at synapses. Our data also show that distinct signaling pathways regulate synaptic and extrasynaptic receptors clustering. Together these results identify a novel role of GSK3β signaling in the regulation of extrasynaptic receptor surface trafficking and GSK3β, PKA and CaMKIIα pathways in facilitating adaptations of synaptic receptors.

Minor comments:

-The authors should increase the number of experiments in figure 7F. It is striking that there is no significant effect of the 4-AP on the diffusion coefficient values.

We have now corrected the figure with new data.

Reviewer #2:

The manuscript advances our knowledge on how the phosphorylation state of gephyrin modulates the GABAA receptor-gephyrin interaction. In particular this is studied in long term plasticity paradigms (48 h). This can contribute clarifying post-synaptic mechanisms of plasticity, a relevant but poorly studied topic.

We are thankful to the reviewer for his/her enthusiasm and recognition of the accomplished work.

Major points:

1) Several sentences are scientifically imprecise or inaccurate through the whole manuscript. Some examples are listed below.

First sentence of the abstract: “Synaptic plasticity relies on the rapid change in neurotransmitter receptor number in the postsynaptic membrane, which is regulated ‐ apart from endocytosis and exocytosis - by lateral diffusion”. While I may understand the message the Authors wants to convey it has to be remarked that synaptic plasticity does not only rely of “rapid change of neurotransmitter receptor number”: While there are example of short term plasticity in which receptor are exchanged in tens of milliseconds, in many forms of plasticity, receptors are rearranged in the time range of minutes Bannai et al., 2009; Petrini et al., 2014).

We have now corrected the text to better explain our message.

Also, the definition “postsynaptic membrane” should be substituted by “postsynaptic sites” or “postsynaptic areas”. Typically it is assumed that “postsynaptic membrane” also includes perisynaptic and extrasynaptic sites.

The reviewer is right. We have done the appropriate modifications.

Introduction, first two lines: “...essential role in information transfer between neuronal networks”: GABAA-mediated information transfer conventionally occurs between two neurons not between networks. If the author really meant that information can be transferred “between neuronal networks” please specify how, and why it is relevant in this context.

Sorry for the confusion, the appropriate changes have been made.

Page 3 line 9 from bottom: “...phosphorylation of PKA...”: as far as I know, phosphorylation of gephyrin (S303) by PKA has not been implicated in the plasticity described in Flores et al., 2015. In that study it is stated that “phosphorylation of gephyrin on S305 site (by CaMKII) is both sufficient and necessary to promote inhibitory synapse formation in response to neuronal activity”. Please clarify.

Corrections have been made.

Page 11, line 11 from bottom: “GABAARs undergo rapid Brownian-type motion at extrasynaptic sites, while...”. This sentence is misleading since it passes the message GABAAR receptors diffuse according the Brownian motion rules only at extrasynaptic sites. Maybe the Authors meant that at extrasynaptic space GABAAR obey to the rules of “free Brownian diffusion” while at synapses GABAAR to those of “confined diffusion”.

We have changed the sentence as following (pg 12 line 3): “GABAARs are known to exhibit faster mobility at extrasynaptic sites as compared with synaptic sites. Due to their interaction with the main scaffolding molecule gephyrin GABAARs are slowed down and confined at synapses. This diffusion-capture of GABAARs is modulated by neuronal activity and constitutes an important basis for synaptic plasticity (ref in (Petrini and Barberis, 2014).”

Page 20, line 7 from bottom: Bannai et al., 2009 did not demonstrate that S327 is involved in calcineurin-dependent dephosphorylation. The paper the Authors are referring to is actually “Wang et al., 2003”, and is only cited in the discussion of Bannai et al., 2009. Please pay attention to the appropriateness of the citations quoted.

Corrections have been made.

Page 21, line 6 from bottom: “After 8 h of 4-AP treatment α2 GABAAR were confined to extrasynaptic locations without influencing the diffusion property of synaptic receptors”. It is obscure how receptors can be confined at extrasynaptic sites since receptors in the extrasynaptic space undergo free diffusion. Did the Author mean that alpha2-containing receptors are actively excluded from the synaptic areas? How can receptors be retained at extrasynaptic sites? Which the forces involved in this “retention” are? Please clarify.

This paragraph has been added (pg 26 line 10): “It became clear with the development of SPT approaches that receptors are also hindered in their diffusion outside synapses via molecular crowding but also through specific protein-protein interactions. A receptor-gephyrin interaction outside inhibitory synapses has been reported earlier (Ehrensperger et al., 2007). GABAARs also colocalize and interact with clathrin-enriched endocytic zones (EZs) that are mostly localized extrasynaptically (Smith et al., 2012). Receptors in EZs don't necessarily undergo internalization. They can be part of a reserve pool of receptors rapidly available upon increase in synaptic activity (Petrini et al., 2014). Conversely, the GABAAR-AP2 interaction within EZs has been shown to indirectly control receptor mobility and number at synapses (Smith et al., 2012).”

2) The term “homeostatic” in some cases is used incorrectly. For instance, in the introduction, the Authors state that homeostatic adaptations take place in Bannai et al., 2009. In contrast that study shows a short term reduction of inhibition following a stimulation protocol (with 4-AP), that has nothing to do with homeostasis. Indeed, it is raised the possibility that transient reduction of inhibition could favor LTP onset, meaning that there is no parallel change between excitability and inhibition (i.e. the definition of homeostatic plasticity). Even less “homeostatic” is the paradigm reported in Petrini et al., 2014 in which, in contrast, they report an anti-homeostatic process where potentiation of inhibition is accompanied by reduction of excitation. Similar concept has been suggested in Marsden et al., 2010.

Bannai et al., 2009 and Petrini et a., 2014 are very good examples of how lateral diffusion tune the synaptic inhibitory strength, but not in a homeostatic way.

The appropriate changes have been made.

My point here is that “homeostatic change” is not a synonym of “synaptic plasticity” or “chronic change”. Homeostatic plasticity is a particular type of plasticity in which synaptic changes maintain the “excitability equilibrium” of neurons or neuronal networks.

We have replaced the misleading term “homeostatic plasticity” by synaptic plasticity.

3) In the introduction, the Authors state that in Saliba et al., 2012 there is enhanced neurotransmission. In contrast, in that study, there is no enhanced neurotransmission but rather increased tonic conductance.

The sentence has been removed.

4) The terms “scaffold” or “scaffolding” are not synonyms of “synaptic clustering of gephyrin”, as they are often used in this manuscript (e.g. page 3, line 7 from top; page 10 line 7 from bottom; page 18 first line).

The reviewer is right. The appropriate changes have been made.

5) The long term effect of 4-AP treatment on gephyrin clustering and GABAAR mobility is opposite with respect to that observed in acute by Bannai et al., 2009. While I think that this “acute” versus “chronic” effect is very interesting, I didn't fully understand the “molecular” explanation provided for this discrepancy. Please clarify.

This is now explained in the discussion (pg 27 line 10) as follows: “Acute 4-AP treatment increases and chronic 4-AP treatment decreases α2 GABAAR lateral diffusion. The observed increase in GABAAR diffusion after acute 4-AP treatment can be explained by increase in synaptic escape of receptors leading to reduced postsynaptic clustering and dispersal of gephyrin molecules away from the synapse (Bannai et al., 2009). On the contrary, we show here that chronic 4-AP treatment leads to synaptic immobilization and recruitment of GABAAR α2 and gephyrin. These discrepancies are probably due to the distinct signaling pathways activated by the acute and chronic changes in activity. Short term 4-AP application induces NMDAR-mediated calcium influx and calcineurin activation leading to dephosphorylation of GABAAR γ2 subunit S327 residue (Bannai et al., 2009). In this context, the relief in GABAAR diffusion constraints arises from receptor dephosphorylation while gephyrin loss is a consequence of receptor dispersal (Niwa et al. 2012). In contrast, we show that chronic changes in activity impacts first the recruitment of gephyrin at synapses, and then allows the recruitment of GABAARs. PKA and CaMKIIα signaling act downstream of NMDAR to facilitate compensatory postsynaptic adaptations at GABAergic synapses (Flores et al., 2015). Our data extends this understanding by demonstrating a role for the GSK3β pathway in addition to PKA and CaMKIIα pathways in facilitating gephyrin scaffold organization of individual GABAARs after prolonged changes in activity.”

6) The S268E and S270A gephyrin synaptic clusters are reported to show different density. The Authors hypothesize that the increased density of S268E clusters is responsible for the reduction in GABAAR mobility at synapses. Although in the Author's view this explanation is rather straightforward, this theory needs to be further discussed in the light of the works that addressed the molecular organization of gephyrin at synapse and/or the stoichiometry gephyrin-GABAAR (e.g. Bannai et al., 2009). Indeed, according to the current theory, gephyrin molecules trimerize through the G domain and, subsequently, trimers dimerize through the E domain to form examers. Thus an “exameric” monolayer latex matrix is formed beneath the synapse. Furthermore, receptors should interact with gephyrin in stoichiometric ratio 1:1. Taking into account these data, how the Authors think an increase of gephyrin could affect the availability of gephyrin molecules at synapses? Indeed according to what is known, gephyrin cannot be further packed (in 2D), unless the authors hypothesize the formation of a different distribution/molecular organization. Alternatively (or in addition) it could be hypothesized a change in the GABAAR-gephyrin stoichiometry. I'm not dogmatic on this, but possible scenarios should be provided (and discussed with respect to previous literature).

The following paragraph has been added to the discussion (pg 25 line 1): “The hexameric gephyrin lattice model was proposed based on G and E domain crystal structures available at the time. However, in recent years atomic force microscopy (AFM) and small-angle X-ray scattering (SAXS) structure of full-length gephyrin has shown that gephyrin only exist as trimers, as individual E domains are in an open extended confirmation (Sander et al., 2013). (Pennacchietti et al., 2017) have shown that after iLTP gephyrin reorganizes itself into distinct subsynaptic nanodomains. Full-length gephyrin can exist in open or closed confirmations based on the linker domain folding (Sander et al., 2013). All the gephyrin phosphorylation sites have been mapped to the linker domain suggesting phosphorylation is a strong candidate for determining open and closed states within gephyrin nanodomains. This could in turn determine the distance between two nanodomains and/or total number of nanodomains within a given synapse.”

7) The authors should better detail the clustering algorithm used for identifying the gephyrin clusters in super-resolution experiments. Does the observed differences of cluster area/density are algorithm-clustering-dependent?

The following paragraph has been added in the Material and methods section (pg 9 line 15): “The threshold to define the border was set to 1000 detections/µm2, taking into account the reported gephyrin densities in synapses (Specht et al, 2013; Fig. 3B). Briefly, all pixels (PALM pixel size=20 nm) containing less than 2 detections where considered as empty, and their intensity value set to zero. The intensity of pixels with at least two detections was set to one. The resulting binary image was analyzed with the function regionprops of Matlab (The Mathworks) to extract the surface area of each cluster identified by this function. Density was calculated as the total number of detections in the pixels belonging to a given cluster, divided the area of the cluster.”

Minor points:

The text must be improved from the grammatical and syntactical point of view. In its present form, the manuscript is difficult to read and the message is obscured. There are also “here and there” some repetitions. I strongly recommend a substantial critical editing of the manuscript by a professional manuscript editor.

The paper has now been edited by a native English speaker.

1) Page 10, line 7 from top: “simulating phosphorylation....”: maybe the authors meant “mimicking phosphorylation”

Changes have been made.

2) Page 11, lines 9 and 10: “similar reduction” and “similar increase”. Specify similar to what.

The sentence was changed as follows (pg 11 line 21): “Similar to the observed changes in eGFP-gephyrin morphology, quantification of cluster intensity for α2 GABAAR showed a significant increase in neurons expressing eGFP-S270A, while eGFP-S268E expressing neurons only showed a modest reduction in α2 (Fig. 1C).”

3) Page 12, line 9 from bottom: “Thus, we concluded the”. Maybe the Authors meant: “Thus we concluded that”. Same issue at page 13, line 10 from bottom

Changes have been made.

4) Page 12 line 5 from bottom: “To test this idea”. Maybe the Authors meant: “To test this hypothesis” Same issue at page 15, line 11

Changes have been made.

5) Page 13 line 4: “then we must also notice”, Although a understand what the author mean I would rephrase it as: “then we should also measure”.

The following changes have been made (pg 13 line 19): “If reducing gephyrin cluster size facilitates α2 diffusion, then we expect shorter dwell time at synaptic sites.”

6) Page 13 last line: “PALM images can indicate”. Instead of “indicate” I would use “resolve”.

Corrected as suggested.

7) Page 15, line 10 from bottom: “increases by 1.95 and 2.3 fold after 8 h or 48 h 4-AP treatment”. Please make “and”/“or” coherent. At the end of the sentence “respectively” should be used.

The following changes were made (pg 16 line 11): “Quantification across independent experiments showed that fluorescence intensity of eGFP-WT gephyrin clusters increases by 1.95 fold after 8 h and by 2.3 fold after 48 h of 4-AP treatment (Fig. 4B).”

8) Page 17, line 10: “Our data identifies”. “Our data identify”.

Corrected as indicated.

9) Page 19, line “: “It is entirely possible”. I would change it with “It is plausible”, for instance.

Corrected as indicated.

10) Page 20, line 10: “determines gephyrin packing”. “Controls” or “modulates”, seem to me more appropriate than “determines”.

Corrected as indicated.
